# Steroid hormones in early pregnancy and adverse birth outcomes: a Chinese birth cohort

**DOI:** 10.3389/fendo.2026.1667039

**Published:** 2026-04-20

**Authors:** Weixiang Wu, Cunwei Ji, Hongyu Li, Fuqiang Diao, Lihong Wu, Xiaolin Ruan, Youwen Luo, Mingyong Luo

**Affiliations:** 1 Department of Clinical Laboratory, Women and Children’s Hospital, Southern University of Science and Technology, Guangzhou, China; 2 Department of Clinical Laboratory, Guangdong Women and Children Hospital, Guangzhou, China

**Keywords:** adverse birth outcomes, androgen, androstenedione, combined effect, steroid hormones, testosterone

## Abstract

**Background:**

Adverse birth outcomes (ABOs) are major public health concerns. While maternal steroid hormones are essential for fetal development, their individual and combined effects on ABO remain unclear.

**Methods:**

This prospective cohort study included 364 pregnant women (30.7 ± 4.0 years) in Guangzhou, China. Nineteen steroid hormones were measured by LC-MS/MS. Regression and Qgcomp models were used to assess individual and combined associations with ABO, including low birth weight, macrosomia, small for gestational age (SGA), large for gestational age, and preterm birth.

**Results:**

Pregnant women with ABO had higher levels of androstenedione (A4) and testosterone (T) and lower levels of 11-deoxycortisol (11-DOF) and estriol (E3). In linear regression models, each 1-SD increase in cortisol (F) was associated with increases of 0.18 cm (95% CI: 0.02, 0.34) in birth length and 0.24 cm (95% CI: 0.04, 0.44) in head circumference, whereas estrone (E1) was inversely associated with gestational age (−0.20 weeks, 95% CI: −0.36, −0.05). In logistic regression analyses, each 1-SD increase in A4 (OR = 1.36, 95% CI: 1.07, 1.72), T (OR = 1.37, 95% CI: 1.08, 1.73), and dihydrotestosterone (DHT) (OR = 1.32, 95% CI: 1.03, 1.69) was associated with higher odds of ABO, whereas 11-DOF was inversely associated with ABO (OR = 0.70, 95% CI: 0.50, 0.98). For SGA, each 1-SD increase in A4 (OR = 1.40, 95% CI: 1.01, 1.94) and T (OR = 1.47, 95% CI: 1.08, 1.99) was associated with increased risk, whereas F (OR = 0.84, 95% CI: 0.72, 0.97) and 11-DOF (OR = 0.58, 95% CI: 0.35, 0.95) were inversely associated. In Qgcomp analyses, the androgen mixture was associated with ABO (OR = 1.40, 95% CI: 1.00, 1.97) and SGA (OR = 1.77, 95% CI: 1.04, 3.01), with A4 and T contributing the largest weights. Consistent directional patterns were observed for androgen-related hormones across outcomes and analytical approaches.

**Conclusions:**

Maternal hormonal milieu in early pregnancy may be associated with fetal growth and ABO risk, with consistent patterns for androgen-related hormones. These findings are exploratory and require validation in larger cohorts.

## Introduction

Adverse birth outcomes (ABO) including low birth weight (LBW), macrosomia, small for gestational age (SGA), large for gestational age (LGA), and preterm birth (PTB), are major public health concerns due to their substantial contribution to neonatal morbidity, mortality, and long-term non-communicable diseases in adulthood (1, 2). Globally, approximately 14.7% of newborns have LBW, and 9.9% are born preterm (3, 4). In China, LBW rates range from 4.6% to 5.7%, with PTB rates steadily increasing over the last 16 years (5, 6). These trends highlight an urgent need for early identification and prevention strategies targeting modifiable risk factors to reduce the public health burden of ABOs. Although the exact causes of ABOs remain unclear, disruptions in maternal endocrine function, particularly involving steroid hormones, are believed to play a vital role.

Steroid hormones, synthesized from cholesterol, rise significantly during pregnancy to regulate placental development, immune regulation, and fetal growth (1, 7). Emerging evidence suggests that hormonal milieu in early pregnancy is closely linked to the risk of ABOs. For instance, progesterone (P4) is crucial for maintaining pregnancy, and low P4 levels in the first trimester have been linked to increased risks of LBW and hypertensive disorders of pregnancy (HDP) (8). Levels of 17α-hydroxyprogesterone (17-OHP4) in the umbilical cord exhibited a gender-specific effect on fetal birth weight, while maternal salivary testosterone (T)/estradiol (E2) ratio has been found to predict fetal growth (9, 10). A recent Mendelian randomization study concluded that higher circulating T levels in females were associated with increased LBW and PTB risk (11). Corticosteroids rise significantly toward the end of gestation in response to the dramatic changes of hypothalamic-pituitary-adrenal (HPA) axis, playing a crucial role in regulating fetal and placental metabolism (1). However, excessive cortisol (F) has been linked to PTB, intrauterine growth restriction, and LBW (12).

Previous human studies have provided important evidence linking maternal androgen exposure to fetal growth and birth outcomes. For example, population-based studies have shown that elevated maternal androgen levels or bioactive androgen activity are associated with reduced birth size and altered fetal growth trajectories (13, 14). However, most existing studies have primarily focused on individual hormones or composite androgen activity, failing to capture the real-world scenario in which pregnant women are simultaneously exposed to multiple steroid hormones. Given that steroid hormones operate within a complex and interconnected regulatory network, the potential synergistic effects of multiple steroid hormones on ABOs remain insufficiently explored, particularly during early pregnancy. We therefore hypothesized that the early pregnancy hormonal milieu, particularly androgen-related hormones, may influence fetal growth and contribute to the risk of ABOs. To address this gap, our study analyzed 19 steroid hormones within a single sample using liquid chromatography-tandem mass spectrometry (LC-MS/MS) in early pregnancy. Both single and multiple hormone models were employed to investigate the individual and combined effects of steroid hormones on ABO risk. Our findings aim to provide insights into the role of the maternal hormonal milieu in early pregnancy and may inform improved risk assessment and early identification of ABOs.

## Materials and methods

### Study population

**Figure 1:**
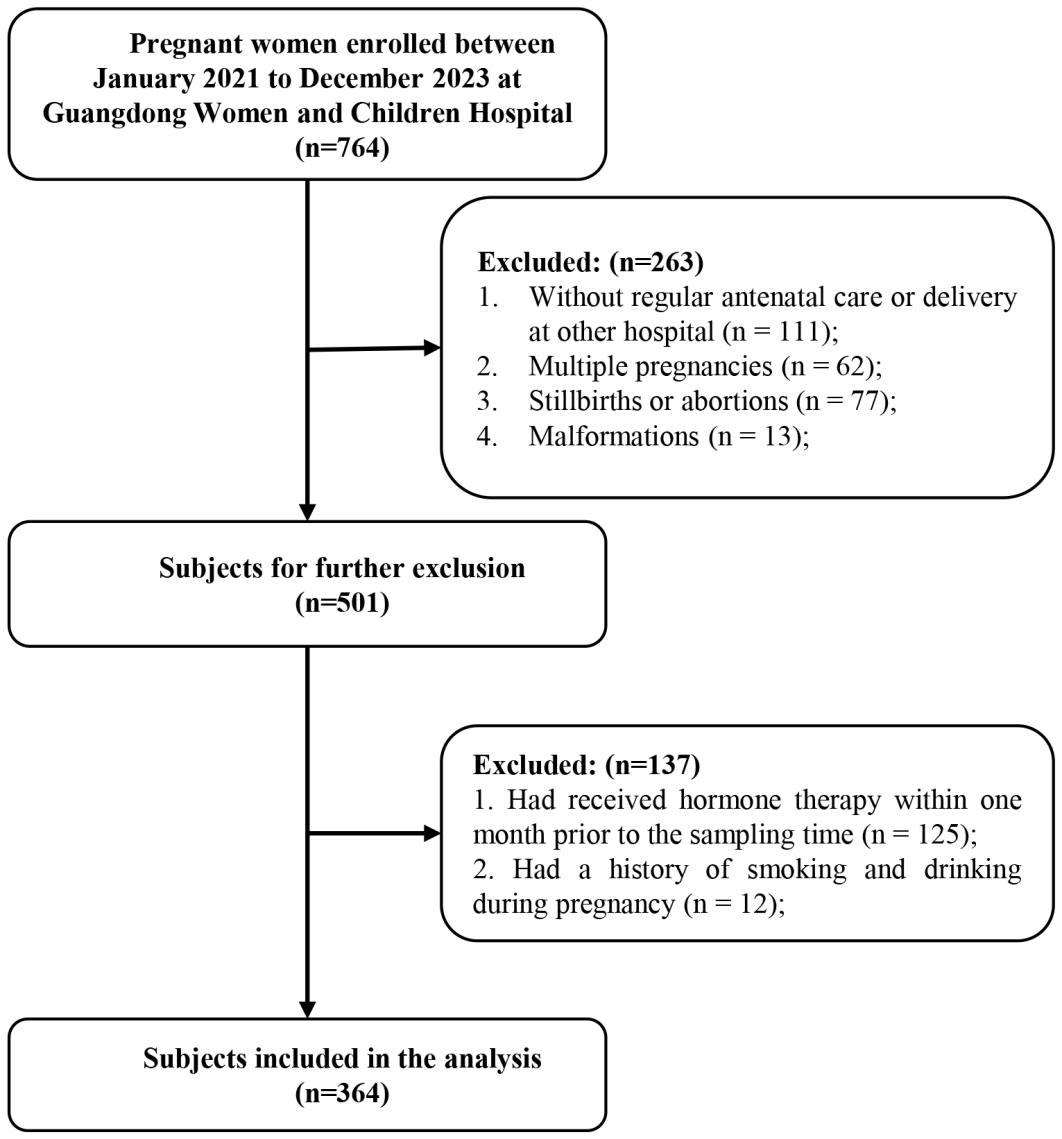
Flow chart of sample selection process.

This prospective birth cohort study was conducted at Guangdong Women and Children Hospital, a large teaching tertiary public hospital in Guangzhou city, Guangdong province, China. Pregnant women attending the study hospital for prenatal examinations between January 2021 and December 2023 were recruited for inclusion in this birth cohort. Eligibility criteria were as follows: (i) natural conception; (ii) gestational age at enrollment ≤ 16 weeks; (iii) maternal age ≥16 years; and (iv) no diagnosed diseases at enrollment, including tumors, autoimmune diseases, heart diseases, infectious diseases, or endocrine disorders (e.g., polycystic ovary syndrome [PCOS] or thyroid disorders). Exclusion criteria were as follows: (i) lack of regular antenatal care; (ii) deliver at other hospital; or (iii) multiple pregnancies, stillbirths, abortions, and malformations. Pregnant women who had received hormone therapy within one month prior to the sampling time were excluded to minimize bias, in consistent with previous studies (9, 15). In addition, 12 pregnant women who reported smoking or alcohol consumption during pregnancy were excluded due to the potential confounding effects of tobacco and alcohol exposure. Finally, a total of 364 healthy pregnant women were included. The selection process for study participants was shown in **Figure 1**. All participants provided informed consent, and the study received ethical approval from the Medical Ethics Committee of Guangdong Women and Children Hospital (No. 202201072). All healthcare procedures were conducted in accordance with approved guidelines and regulations.

### Sample collection, data extraction and ABO definitions

With participants’ informed consent, professional physicians collected 5 ml of venous blood at the time of enrollment (14.22 ± 1.51 weeks) using coagulation-promoting tubes. This gestational window corresponds to the early pregnancy period when placental steroidogenesis becomes established, allowing assessment of maternal steroid hormone levels (7). Samples were centrifuged at 3000 r/min for 10 min, and serum was separated into cryovials and stored in a −80 °C freezer. The maternal basic information and anthropometric data of newborns were extracted either from hospital medical records or obtained through face-to-face questionnaires administered by experienced nurses. Maternal information included maternal age, pre-pregnancy BMI, education level, delivery mode, parity, gravidity, smoking and drinking habits, pregnancy complications (e.g. HDP and gestational diabetes [GDM]). After delivery, the obstetric nurses promptly measured and recorded the birth weight (BW, g), length (BL, cm), head circumstance (HC, cm), and gestational age (GA, week) of the newborns. Pre-pregnancy BMI was calculated as the ratio of pre-pregnancy weight to the square of height. According to World Health Organization (WHO) criteria, pre-pregnancy BMI were assigned as underweight (< 18.5 kg/m^2^), normal weight (18.5 to 24.9 kg/m^2^), overweight (≥ 25.0 kg/m^2^), respectively.

ABO women were those delivering babies with LBW, macrosomia, small for gestational age (SGA), large for gestational age (LGA), or PTB. LBW was defined as a BW < 2500 g, and macrosomia as a BW ≥ 4000 g. Based on a reference of population-based GA-specific BW percentiles for contemporary Chinese, BWs were categorized into SGA (BW ≤ the sex-specific 10th percentile for GA) and LGA (BW ≥ the sex-specific 90th percentile for GA) (16). PTB was defined as a GA < 37 weeks. No post-term birth was included in this study.

### Steroid hormone analyses

A total of 19 steroid hormones were measured to represent key intermediates and end-products of the steroidogenesis pathway, covering major steroid classes. These hormones were selected *a priori* based on established biological pathways and prior literature (7, 17, 18). Specifically, the panel included 5 glucocorticoids (F, cortisone [E], corticosterone [CORT], 11-deoxycortisol [11-DOF], 21-deoxycortisol [21-DOF]), 2 mineralocorticoids (aldosterone [ALD], 11-deoxycorticosterone [DOC]), 5 androgens (androstenedione [A4], T, dihydrotestosterone [DHT], dehydroepiandrosterone [DHEA], dehydroepiandrosterone sulfate [DHEAS]), 3 estrogens (estrone [E1], E2, estriol [E3]), and 4 progestogens (P4, pregnenolone [P5], 17-OHP4, 17-hydroxypregnenolone [17-OHP5]). This method, developed based on a previously described method (18), was conducted using an ExionLC AC liquid chromatograph coupled with a 6500 Qtrap LC-MS/MS system (AB SCIEX, Toronto, Canada). Details of the sample analysis and quality assurance are provided in the **Supplementary Information (Methods**; **Supplementary Tables S1**; **S2**). The coefficient of variation ranged from 4.20% to 11.93%, and the limit of quantitation (LOQ) ranged from 0.003 to 0.407 ng/mL (**Supplementary Table S3**). Seventeen hormones had a 100% detection rate, E3 had 94%, and 17-OHP5 had 68.4%. For concentrations below the LOQ, a value of LOQ/√2 was assigned.

### Statistical analyses

No missing data were observed for the variables included in the analyses; therefore, all analyses were conducted using complete data. The general characteristics of participants are presented as mean ± SD or median (IQR) for continuous variables, and frequencies (%) for categorical data. The distribution of 19 steroid hormones in pregnant women is shown as geometric mean, mean, and percentiles. Participants were grouped by maternal age (non-AMA: age <35 vs. AMA: age ≥35), pre-pregnancy BMI (underweight, normal, overweight), and ABO (ABO vs. non-ABO). Group differences were compared using Mann-Whitney or Kruskal-Wallis tests. Steroid hormone concentrations were ln-transformed to reduce skewness for Pearson correlation analyses.

Multiple regression models were employed to examine the associations between maternal steroid hormone levels, birth parameters, and ABO risk. Covariates were selected *a priori* based on previous literature and biological plausibility, and the final adjustment sets are provided in **Supplementary Table S4**. Hormone concentrations were standardized using z-scores, and effect estimates are reported per 1-SD increase with corresponding 95% CIs to facilitate clinical interpretation. For linear regression models evaluating continuous birth parameters, models were adjusted for maternal age, education, pre-pregnancy BMI, delivery mode, parity, HDP, GDM, and infant sex for GA. Because GA is a major determinant of fetal growth, models for BW, BL, and HC were additionally adjusted for GA. For logistic regression models, cases were defined as pregnancies with LBW, SGA, macrosomia, LGA, and PTB, whereas controls were pregnancies without any ABOs. Models for ABO, SGA, and LGA were adjusted for sampling gestational week, maternal age, education, pre-pregnancy BMI, delivery mode, parity, HDP, GDM, and infant sex. GA was additionally adjusted for in LBW and macrosomia models, but not in SGA/LGA models, as these outcomes are defined using GA-specific percentiles, or in PTB models, where GA is inherent to the outcome definition. False discovery rate (FDR) correction was performed using the Benjamini–Hochberg procedure.

Quantile g-computation (Qgcomp), implemented using the *qgcomp* package in R, was applied to evaluate the joint effects of multiple steroid hormone exposures on the risk of ABOs. Ln-transformed hormone concentrations were included in the Qgcomp models to account for skewed distributions. In addition to the overall steroid mixture, class-specific Qgcomp analyses were conducted according to biologically defined hormone groups. The same covariate adjustment strategy used in the logistic regression models was applied to the Qgcomp analyses. Sensitivity analyses were conducted to assess the consistency of the findings. Regression models were repeated using ln-transformed hormone concentrations. To evaluate the influence of collinearity and model specification in the Qgcomp analyses, models were refitted after excluding highly correlated hormones (*r* > 0.90) and after removing A4, which showed a dominant weight in the androgen-specific model. In addition, regression and Qgcomp analyses were repeated after excluding participants with HDP or GDM. All analyses were performed conducted using R software (version 4.2.2; R Foundation for Statistical Computing, Vienna, Austria). Statistical significance was set at *P* < 0.05 (two-tailed).

## Results

### Basic characteristics of the study population

A total of 364 pregnant women were included in the present study. The average maternal age and pre-pregnancy BMI was 30.7 ± 4.0 years and 21.2 ± 3.0 kg/m^2^ (**Supplementary Table S5**). The prevalence rate of ABO was 22.5% (n = 82), including 14 cases of LBW, 32 of SGA, 11 of macrosomia, 24 of LGA, and 28 of PTB. Characteristics of study subjects according to ABO group are summarized in **Table 1**. Except for GA, BW, BL, and HC, the distribution of other basic demographic characteristics between the subjects with and without ABO showed no statistically significant differences (all *P* > 0.05).

**Table 1:** Characteristics of study subjects according to ABO group.

Characteristics	Non-ABO group (n = 282)	ABO group (n = 82)	*P*-value
Maternal age (year)	30.7 ± 3.8	30.8 ± 4.7	0.590
Pre-pregnancy BMI (kg/m^2^)	21.1 ± 3.0	21.4 ± 3.1	0.606
Pre-pregnancy BMI categories			0.667
Underweight (< 18.5 kg/m2)	41 (14.5)	9 (11.0)	
Normal-weight (18.5-23.9 kg/m2)	196 (69.5)	58 (70.7)	
Overweight (>23.9 kg/m2)	45 (16.0)	15 (18.3)	
Level of education			
Less than bachelor degree	66 (23.4)	26 (31.7)	0.253
Bachelor degree or above	216 (76.6)	56 (68.3)	
Mode of delivery			0.056
Vaginal delivery	184 (65.2)	44 (53.7)	
Cesarean section	98 (34.8)	38 (46.3)	
Nulliparous	143 (50.7)	48 (58.5)	0.212
Hypertension during pregnancy	7 (2.5)	2 (2.4)	0.982
Gestational diabetes	45 (16.0)	17 (20.7)	0.311
Gestational age at sampling (week)	14.2 ± 1.5	14.1 ± 1.5	0.498
Male infant	151 (53.5)	48 (58.5)	0.424
Birth parameter			
GA (week)	39.4 ± 0.9	38.1 ± 1.9	<0.001
BW (g)	3246.6 ± 260.3	3002.9 ± 679.0	<0.001
BL (cm)	49.6 ± 1.2	48.7 ± 2.8	<0.001
HC (cm)	33.7 ± 1.0	33.3 ± 2.3	<0.001

SD, standard deviation; BMI, body mass index; GA, Gestational age at delivery; BW, birth weight; BL, birth length; HC, head circumference. Continuous variables are presented as mean ± SD, and categorical variables are presented as n (%).

### Distribution characteristics of 19 steroid hormones in the early pregnancy

**Figure 2:**
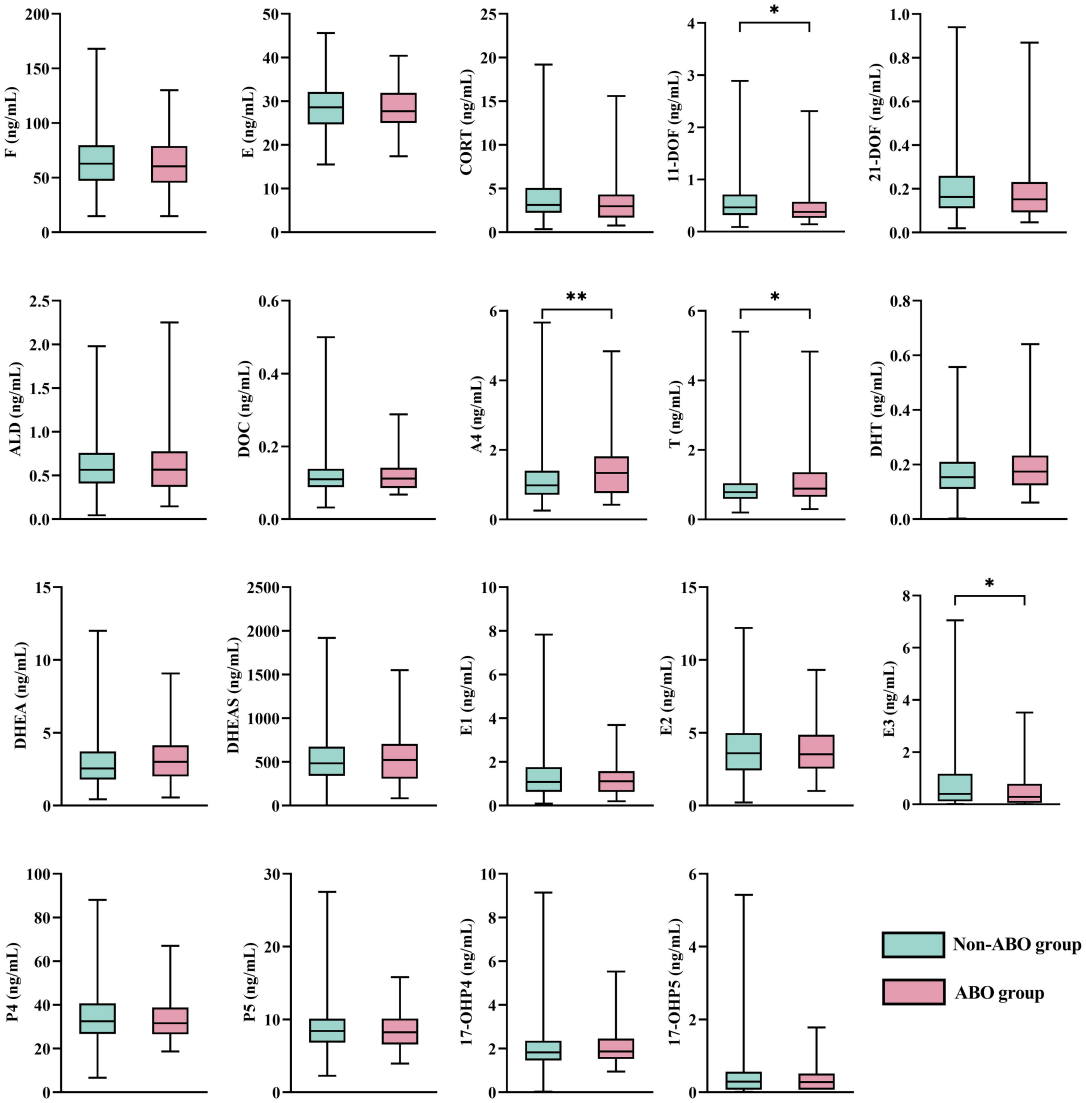
Distributions of 19 steroid hormones in pregnant women according to ABO. Data are presented as box plots showing the median (line), interquartile range (box), and range (whiskers). The green color indicates the non-ABO group, and the red color indicates the ABO group. Differences between groups were assessed using the Mann–Whitney U test. ^*^P < 0.05, ^**^P < 0.01.

The distribution characteristics of 5 glucocorticoids, 2 mineralocorticoids, 5 androgens, 3 estrogens, and 4 progestogens are presented in **Table 2**. Among the overall study population, the median concentration of DHEAS was the highest (496.0 ng/mL), while the median concentration of DOC was the lowest (0.11 ng/mL). E3 and 17-OHP5 showed relatively wide concentration ranges, including values below the LOQ, reflecting their skewed distributions and biological variability. As shown in **Figure 2** and **Supplementary Table S6**, when stratified by ABO status, pregnant women with ABO had significantly higher levels of A4 (1.34 vs. 0.99 ng/mL) and T (0.89 vs. 0.78 ng/mL), but lower levels of 11-DOF (0.38 vs. 0.47 ng/mL) and E3 (0.29 vs. 0.40 ng/mL). In the AMA group, A4 (0.83 vs. 1.05 ng/mL), DHT (0.13 vs. 0.16 ng/mL), and DHEAS (404.5 vs. 511.0 ng/mL) were significantly lower (**Supplementary Figure S1**). A4, T, DHT, DHEA, and DHEAS levels were negatively associated with maternal age (all *P* < 0.01, **Supplementary Figure S2**). Among BMI categories, DHEAS (*P* = 0.001) and P4 (*P* < 0.001) levels differed significantly, with DHEAS increasing and P4 decreasing with higher pre-pregnancy BMI (**Supplementary Figure S3**). Each BMI category raised DHEAS by 106.87 ng/mL (*P* < 0.001) and reduced P4 by 3.90 ng/mL (*P* < 0.001). Pearson correlation analysis (**Supplementary Figure S4**) showed generally positive correlations among most hormones (*r* = 0.10–0.98), with the strongest between CORT and 21-DOF (*r* = 0.98). Moderate-to-strong correlations (*r* = 0.21–0.85) were also observed among androgen-related hormone.

**Table 2:** Distribution characteristics of 19 steroid hormones in early pregnancy (n = 364, ng/mL).

Type	Steroid hormone	GM	Mean	25^th^	50^th^	75^th^	Range
Glucocorticoid	F	60.0	65.2	47.1	62.1	79.7	14.7−158.0
	E	28.0	28.6	24.8	28.6	32.1	15.5−45.6
	CORT	3.25	4.04	2.20	3.11	4.95	0.36−19.2
	11-DOF	0.46	0.55	0.31	0.45	0.69	0.09−2.89
	21-DOF	0.17	0.21	0.11	0.16	0.25	0.02−0.94
Mineralocorticoid	ALD	0.55	0.62	0.40	0.56	0.76	0.05−2.25
	DOC	0.11	0.12	0.09	0.11	0.14	0.03−0.50
Androgen	A4	1.06	1.23	0.73	1.03	1.51	0.26−5.66
	T	0.83	0.96	0.60	0.80	1.09	0.20−5.40
	DHT	0.15	0.17	0.11	0.16	0.21	0.02−0.64
	DHEA	2.60	3.01	1.82	2.68	3.83	0.43−12.0
	DHEAS	459.0	536.3	324.5	496.0	680.5	2.04−1420.0
Estrogen	E1	1.04	1.34	0.63	1.09	1.73	0.09−7.83
	E2	3.41	3.88	2.46	3.60	4.93	0.21−12.2
	E3	0.29	0.76	0.11	0.37	1.06	<LOQ−7.05
Progestogen	P4	32.6	34.2	26.6	32.4	40.1	6.66−88.1
	P5	8.23	8.63	6.75	8.40	10.1	2.25−27.5
	17-OHP4	1.85	2.02	1.46	1.83	2.38	0.08−9.14
	17-OHP5	0.26	0.48	<LOQ	0.29	0.56	<LOQ−5.42

GM, geometric mean; 25^th^, 50^th^, and 75^th^ are the percentiles; LOQ, limit of quantitation.

### Association of steroid hormone levels in early pregnancy with birth parameters

**Table 3** shows the association between ln-transformed hormone levels and BW, BL, HC, and GA. A positive association of F levels in relation to length and HC at birth was observed. For each 1-SD increase in F levels (25.78 ng/mL), BL increased by 0.18 (95% CI: 0.02, 0.34) cm and HC increased by 0.24 (95% CI: 0.04, 0.44) cm. Conversely, a decreasing trend of E1 levels associated with GA was suggested, and per SD-increase in E1 (1.07 ng/mL) might reduce GA by 0.20 (95% CI: −0.36, −0.05) week.

**Table 3:** Multiple linear models on the association between steroid hormones (per 1-SD increase) and birth parameters.

Steroid Hormones (SD, ng/mL)	BW (g)		BL (cm)		HC (cm)		GA (week)	
	β (95% CI)	*P*-value	β (95% CI)	*P*-value	β (95% CI)	*P*-value	β (95% CI)	*P*-value
F (25.78)	35.28 (−1.44, 72.00)	0.061	0.18 (0.02, 0.34)	0.032	0.24 (0.04, 0.44)	0.017	−0.07 (−0.22, 0.08)	0.339
E (5.62)	30.17 (−5.01, 65.36)	0.094	0.09 (−0.06, 0.25)	0.239	0.05 (−0.09, 0.18)	0.483	−0.04 (−0.18, 0.10)	0.568
CORT (3.09)	33.53 (−1.32, 68.38)	0.060	0.11 (−0.04, 0.27)	0.149	0.11 (−0.02, 0.24)	0.099	0.04 (−0.10, 0.18)	0.566
11-DOF (0.36)	17.56 (−18.18, 53.31)	0.336	0.00 (−0.15, 0.16)	0.951	0.01 (−0.13, 0.14)	0.940	0.06 (−0.09, 0.20)	0.442
21-DOF (0.16)	32.28 (−2.70, 67.25)	0.071	0.11 (−0.04, 0.27)	0.150	0.11 (−0.02, 0.24)	0.105	0.05 (−0.09, 0.19)	0.503
ALD (0.3)	2.71 (−32.03, 37.45)	0.879	0.05 (−0.10, 0.20)	0.541	−0.07 (−0.20, 0.06)	0.276	−0.02 (−0.15, 0.12)	0.819
DOC (0.05)	12.84 (−21.87, 47.54)	0.469	0.07 (−0.08, 0.22)	0.384	0.04 (−0.09, 0.17)	0.541	−0.12 (−0.26, 0.02)	0.093
A4 (0.78)	4.16 (−30.81, 39.14)	0.816	0.04 (−0.11, 0.19)	0.622	−0.06 (−0.19, 0.07)	0.356	−0.04 (−0.18, 0.10)	0.542
T (0.62)	6.69 (−27.97, 41.35)	0.706	0.04 (−0.11, 0.19)	0.582	−0.01 (−0.15, 0.12)	0.822	−0.07 (−0.20, 0.07)	0.355
DHT (0.09)	−3.91 (−39.18, 31.36)	0.828	0.01 (−0.15, 0.16)	0.910	−0.07 (−0.20, 0.06)	0.306	−0.12 (−0.26, 0.02)	0.084
DHEA (1.67)	19.52 (−15.15, 54.18)	0.271	0.06 (−0.09, 0.21)	0.432	0.01 (−0.12, 0.14)	0.878	−0.07 (−0.21, 0.07)	0.312
DHEAS (284.49)	−15.62 (−51.70, 20.45)	0.396	−0.02 (−0.18, 0.13)	0.765	−0.11 (−0.25, 0.02)	0.099	−0.07 (−0.21, 0.08)	0.372
E1 (1.07)	14.93 (−21.69, 51.54)	0.425	0.07 (−0.09, 0.23)	0.409	−0.03 (−0.16, 0.11)	0.705	−0.20 (−0.36, −0.05)	0.010
E2 (1.95)	22.29 (−16.54, 61.13)	0.261	0.07 (−0.10, 0.24)	0.402	−0.05 (−0.20, 0.09)	0.493	−0.07 (−0.22, 0.09)	0.384
E3 (0.98)	17.39 (−20.65, 55.42)	0.371	0.07 (−0.10, 0.24)	0.410	0.04 (−0.10, 0.19)	0.550	−0.02 (−0.17, 0.13)	0.769
P4 (10.59)	−2.04 (−38.04, 33.96)	0.912	0.00 (−0.16, 0.15)	0.991	−0.07 (−0.20, 0.06)	0.305	0.02 (−0.12, 0.17)	0.735
P5 (2.75)	−0.10 (−34.65, 34.45)	0.996	0.03 (−0.12, 0.18)	0.682	0.03 (−0.10, 0.16)	0.618	0.00 (−0.13, 0.14)	0.945
17-OHP4 (0.95)	14.28 (−20.26, 48.82)	0.418	0.06 (−0.09, 0.21)	0.420	0.04 (−0.09, 0.17)	0.568	−0.12 (−0.26, 0.02)	0.082
17-OHP5 (0.64)	27.81 (−6.95, 62.58)	0.118	0.05 (−0.11, 0.20)	0.542	0.07 (−0.06, 0.20)	0.314	0.06 (−0.08, 0.20)	0.422

Hormone concentrations were standardized using z-score transformation. Models were adjusted for sampling gestational week, maternal age, education level, pre-pregnancy BMI, delivery mode, parity, HDP, GDM, and infant sex for GA, with GA additionally adjusted for BW, BL, and HC.

### Association of steroid hormone levels in early pregnancy with ABO risk

**Table 4** and **Supplementary Table S7** show the associations between steroid hormones and risks for all ABOs and individual ABOs. In fully adjusted logistic models, A4, T, and DHT levels in early pregnancy were associated with higher ABO risk, with each 1-SD increase linked to a 1.36-fold (95% CI: 1.07, 1.72), 1.37-fold (95% CI: 1.08, 1.73), and 1.32-fold (95% CI: 1.03, 1.69) increase, respectively. In contrast, 11-DOF levels were inversely associated with ABO risk, with each 1-SD increase (0.36 ng/mL) corresponding to a 0.70-fold lower risk (OR = 0.70, 95% CI: 0.50, 0.98). For individual ABO outcomes, A4 and T were associated with increased SGA risk, with each 1-SD increase linked to a 1.40-fold (95% CI: 1.01, 1.94) and 1.47-fold (95% CI: 1.08, 1.99) higher risk, respectively. Conversely, F and 11-DOF showed inverse associations with SGA, with each 1-SD increase associated with a 0.84-fold (95% CI: 0.72, 0.97) and 0.58-fold (95% CI: 0.35, 0.95) risk, respectively. No clear associations were observed for LBW, macrosomia, LGA, or PTB with hormone levels in early pregnancy (**Supplementary Table S7**, all *P* > 0.05). After FDR correction, none of the associations between maternal steroid hormones and birth parameters or outcomes remained statistically significant (**Supplementary Table S8**, all FDR-adjusted *P* > 0.05); however, consistent overall patterns were observed, particularly for androgen-related hormones.

**Table 4:** Multiple logistic models on the association between steroid hormones (per 1-SD increase) and ABO, SGA, and LGA risk.

Steroid Hormones (SD, ng/mL)	ABO		SGA		LGA	
	OR (95% CI)	*P*-value	OR (95% CI)	*P*-value	OR (95% CI)	*P*-value
F (25.78)	0.88 (0.66, 1.16)	0.356	0.84 (0.72, 0.97)	0.021	1.05 (0.62, 1.78)	0.850
E (5.62)	0.92 (0.71, 1.20)	0.558	0.88 (0.59, 1.31)	0.533	1.07 (0.67, 1.72)	0.776
CORT (3.09)	0.84 (0.62, 1.13)	0.246	0.69 (0.42, 1.15)	0.158	1.09 (0.70, 1.70)	0.704
11-DOF (0.36)	0.70 (0.50, 0.98)	0.037	0.58 (0.35, 0.95)	0.032	0.53 (0.25, 1.11)	0.091
21-DOF (0.16)	0.85 (0.63, 1.15)	0.283	0.73 (0.46, 1.18)	0.197	1.10 (0.70, 1.72)	0.677
ALD (0.3)	1.11 (0.86, 1.44)	0.414	1.22 (0.85, 1.74)	0.276	1.03 (0.60, 1.78)	0.910
DOC (0.05)	0.96 (0.74, 1.24)	0.735	0.83 (0.52, 1.33)	0.441	1.10 (0.71, 1.70)	0.671
A4 (0.78)	1.36 (1.07, 1.72)	0.012	1.40 (1.01, 1.94)	0.042	1.42 (0.97, 2.10)	0.074
T (0.62)	1.37 (1.08, 1.73)	0.009	1.47 (1.08, 1.99)	0.013	1.32 (0.95, 1.82)	0.099
DHT (0.09)	1.32 (1.03, 1.69)	0.027	1.28 (0.90, 1.83)	0.168	1.57 (0.99, 2.49)	0.053
DHEA (1.67)	1.19 (0.94, 1.53)	0.154	1.25 (0.88, 1.78)	0.208	1.22 (0.81, 1.85)	0.341
DHEAS (284.49)	1.10 (0.85, 1.43)	0.477	1.34 (0.90, 1.98)	0.153	0.86 (0.51, 1.44)	0.558
E1 (1.07)	0.91 (0.67, 1.23)	0.539	0.80 (0.50, 1.27)	0.345	0.99 (0.58, 1.69)	0.980
E2 (1.95)	1.03 (0.77, 1.38)	0.846	1.11 (0.74, 1.67)	0.621	0.91 (0.51, 1.64)	0.764
E3 (0.98)	0.75 (0.53, 1.07)	0.109	0.79 (0.49, 1.28)	0.340	0.59 (0.28, 1.25)	0.167
P4 (10.59)	1.05 (0.80, 1.36)	0.737	1.06 (0.72, 1.56)	0.759	0.98 (0.57, 1.67)	0.941
P5 (2.75)	0.97 (0.75, 1.25)	0.796	1.03 (0.71, 1.51)	0.869	1.03 (0.66, 1.60)	0.911
17-OHP4 (0.95)	1.00 (0.77, 1.28)	0.975	0.81 (0.49, 1.31)	0.387	1.17 (0.78, 1.74)	0.447
17-OHP5 (0.64)	0.81 (0.58, 1.13)	0.211	0.73 (0.43, 1.23)	0.232	1.02 (0.64, 1.63)	0.929

Hormone concentrations were standardized using z-score transformation. Models were adjusted for sampling gestational week, maternal age, education level, pre-pregnancy BMI, delivery mode, parity, HDP, GDM, and infant sex.

### Combined effects of steroid mixtures on ABO risk

**Figure 3:**
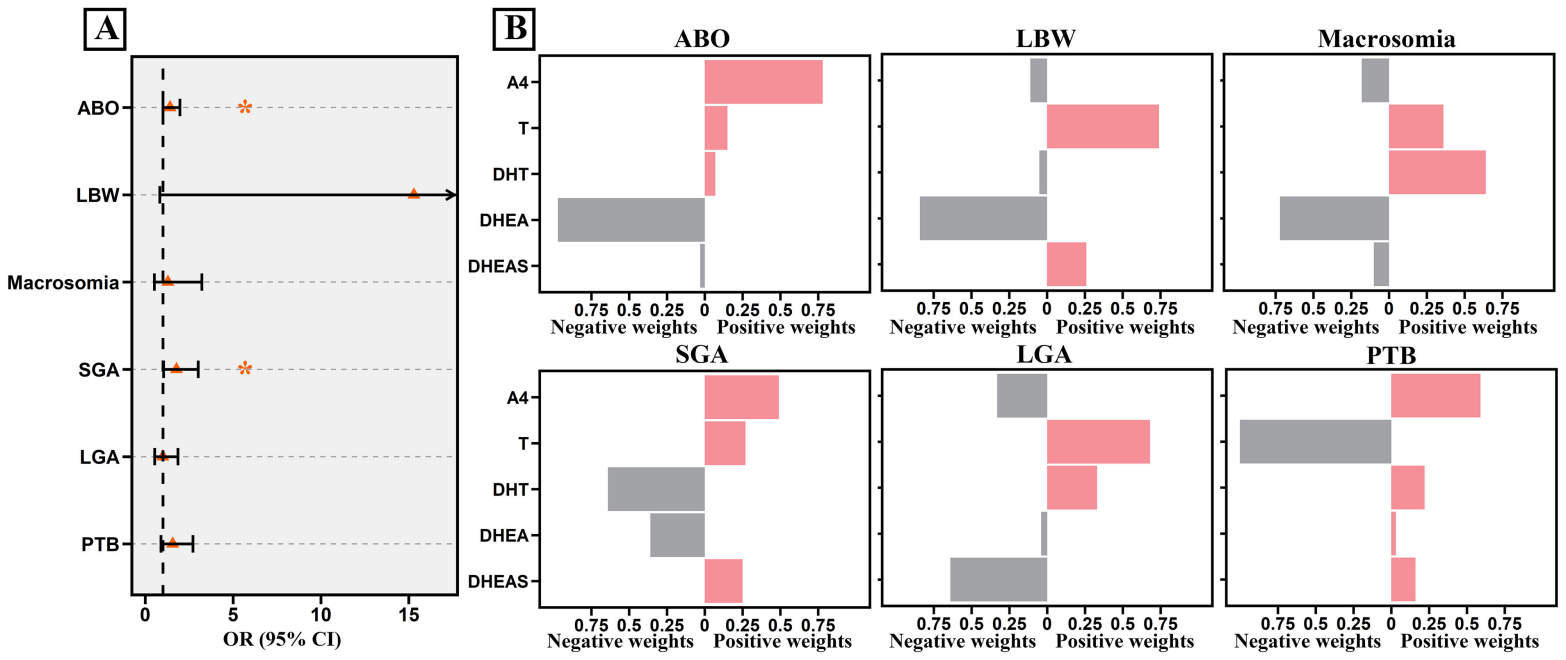
Effects of a mixture of five androgens on ABOs based on Qgcomp model analyses. **(A)** Combined effect of the androgen mixture on ABOs, presented as ORs with 95% CIs. **(B)** Contributions of each androgen on ABOs. The Qgcomp models were adjusted for sampling gestational week, maternal age, education level, pre-pregnancy BMI, delivery mode, parity, HDP, GDM, and infant sex for ABO, SGA, and LGA, with GA additionally adjusted for LBW and macrosomia. The red bars represent positive weights, while the gray bars represent negative weights. ^*^P < 0.05.

In this study, the combined effects of steroid mixtures during pregnancy on ABO risk were explored using the Qgcomp model. As shown in **Supplementary Figure S5**, the overall effect of the total steroid mixture on ABO risk was not statistically significant (OR = 0.71, 95% CI: 0.38, 1.32). Among the individual components, A4 contributed the largest positive weight (0.31), while 11-DOF had the largest negative effect (−0.30). Similarly, no significant combined effects of the total steroid mixture were observed for LBW, macrosomia, SGA, LGA, or PTB (all *P* > 0.05). Although the overall steroid mixture was not significantly associated with ABO, class-specific analyses were further examined given the biological heterogeneity of steroid hormone subclasses. The combined effects of five categories of steroid hormones were further analyzed. As shown in **Figure 3**, the androgen mixture exhibited a positive effect on ABO risk (OR = 1.40, 95% CI: 1.00, 1.97) and SGA risk (OR = 1.77, 95% CI: 1.04, 3.01). Consistent patterns of association were observed for androgen-related hormones across analytical approaches, A4 and T showed the largest positive weights for both ABO risk (0.78 for A4 and 0.15 for T) and SGA risk (0.49 for A4 and 0.27 for T), in line with their positive associations in the regression analyses. No significant associations were found for the other categories of steroid hormones (see **Supplementary Figures S6**−**S9**).

### Sensitivity analyses

Sensitivity analyses showed generally consistent patterns of association (**Supplementary Tables S9**–**S15**). Results based on ln-transformed hormone concentrations were similar in direction to the primary analyses. Analyses excluding participants with HDP or GDM yielded comparable patterns, although some associations were attenuated and no longer statistically significant. In Qgcomp models, excluding highly correlated hormones or removing A4 did not materially change the direction of associations. Overall, androgen-related hormones showed consistent directional associations with ABO and SGA across sensitivity analyses.

## Discussion

This prospective study suggests that maternal hormonal milieu in early pregnancy may be associated with fetal growth and ABO risk. Notably, consistent patterns were observed for androgen-related hormones (A4, T, and DHT), which were positively associated with ABO and SGA across multiple analyses, while 11-DOF showed inverse associations. In addition, F was positively associated with BL and HC, whereas E1 was inversely associated with GA. Qgcomp analysis further supported this pattern, with the androgen mixture associated with ABO and SGA and A4 and T contributing the largest weights. These findings highlight a consistent pattern of association across analytical approaches rather than isolated significant results.

Androgens play an important role in fetal growth and development, although their effects during pregnancy have been less extensively studied than those of estrogens and progestogens (19, 20). In the present study, androgen-related hormones showed consistent patterns of association across both single-hormone and mixture-based analyses, with higher levels of A4 and T associated with impaired fetal growth and increased risks of SGA and ABOs. Notably, these associations were more evident for SGA than for LBW or PTB, which may reflect differences in underlying biological mechanisms. While LBW is a heterogeneous outcome and PTB primarily reflects gestational timing, SGA specifically indicates impaired fetal growth (21). This is consistent with evidence that fetal growth restriction is largely driven by placental dysfunction, suggesting that androgen-related hormones may preferentially influence placental function and fetal growth rather than the timing of delivery (22, 23). Our findings are in line with previous studies reporting associations between maternal androgen exposure and fetal growth. For example, elevated A4 has been observed in SGA offspring (19), and elevated maternal T levels have been linked to growth restriction *in utero* (24). Other studies have also reported associations between maternal androgen levels and neonatal anthropometric measures, supporting a role of the maternal androgen milieu in fetal growth regulation (11, 13, 14). The hormone levels observed in our cohort were broadly comparable to those reported in general pregnant populations and did not indicate pathological hyperandrogenism, suggesting that these associations may occur within the physiological range (25). This differs from conditions such as PCOS, where markedly elevated androgen levels are often accompanied by metabolic dysfunction and are associated with broader pregnancy complications (26, 27). Elevated androgens have been shown to disrupt trophoblast invasion, placentation, and fetal nutrient delivery, providing biological plausibility for their association with impaired fetal growth (28). Animal studies further support that excess maternal androgens disrupt placental growth and metabolism, providing biological plausibility for impaired fetal growth (23). From a clinical perspective, these findings suggest that maternal androgen profiles during early pregnancy may have potential value for identifying pregnancies at risk of impaired fetal growth; however, these findings should be interpreted as exploratory and require further validation before clinical application.

Simultaneously measuring 19 steroid hormones in the same sample provides a comprehensive understanding of the hormonal environment, enabling analysis of both individual and combined effects. Single-hormone regression models often overlook hormonal interactions, such as synergistic or antagonistic effects, potentially contributing to discrepancies in previous studies (19, 20, 24). In this study, the Qgcomp model supported the overall pattern of positive associations for androgen-related hormones (particularly A4 and T) with ABO and SGA, consistent with the multivariable regression analyses. Hormone ratios, like P4/E2, assess hormone balance but are limited to two-hormone interactions (27). Dimensionality reduction methods such as orthogonal partial least squares discriminant analysis (OPLS-DA) and principal component analysis help reduce complexity and identify group differences in steroid profiles. For example, Yuan et al. utilized OPLS-DA to identify 11-DOF as a novel biomarker for trisomy 21 (29). However, analyzing combined effects provides a more complete view of how multiple hormones interact to influence outcomes.

While mixture analyses are common in studies of vitamins, nutrients, and synthetic steroids (30, 31), most human studies of maternal androgens have focused on individual hormones or overall androgen activity (13, 14). In this context, our study applied a mixture-based approach to evaluate the joint exposure to five androgens (A4, T, DHT, DHEA, and DHEAS) during pregnancy and found that combined androgen exposure was associated with increased risks of SGA and ABO. Taken together, these findings suggest a consistent pattern of association for androgen-related hormones across analytical approaches, rather than isolated findings. This insight can guide the development of better screening tools for at-risk pregnancies, enabling timely interventions. Maternal age and pre-pregnancy BMI were also identified as significant factors influencing androgen levels, and these factors should be considered when interpreting our findings. Animal studies are needed to validate the mechanisms behind these combined androgen effects and their potential use in prenatal care.

During pregnancy, estrogens undergo significant changes to support fetal development. E1 and E2 are produced in the ovaries, adipose tissue, and placenta, while E3 is primarily synthesized from fetal precursors and produced at higher rates during pregnancy (32). Our study found that elevated E1 levels tended to reduce GA at delivery, but showed no association with ABOs. In contrast, E3 demonstrated a protective effect against ABOs. While E1 may indicate maternal endocrine disorders, its clinical value during pregnancy remains less clear. E3, however, has long been used as a marker of fetal health, with low levels indicating increased risks for fetal growth restriction and other complications (32). Our findings align with Settiyanan et al., who reported that low E3 levels increase the risk of FGR and LBW, suggesting a protective role for E3 (33). Similarly, Olsen et al. found elevated E3 levels correlated with longer GA and increased PTB risk (34), while Jelliffe-Pawlowski et al. found no association with PTB (35). Overall, these findings suggest a potential but not entirely consistent pattern for estrogen-related hormones, warranting cautious interpretation.

Glucocorticoids, especially F, regulate glucose metabolism, blood pressure, and inflammation (1). During pregnancy, F levels rise through HPA axis and placental regulation. In our study, early pregnancy F levels were positively associated with HC and inversely associated with SGA risk. However, the magnitude of the association was modest, and its clinical significance should be interpreted cautiously. While some studies link higher prenatal F to LBW/SGA (12, 36), other researches have not demonstrated this association (37, 38). In line with our findings, Braithwaite et al. demonstrated in a cohort study that maternal prenatal F levels were positively associated with BW in a sex-specific manner (38). Our F levels (median: 62.8 ng/mL; range: 14.7–158.0 ng/mL) were lower and less variable than those reported in previous studies (36), which may reflect the use of LC-MS/MS with higher analytical specificity compared to earlier immunoassay-based methods (39). Additionally, 11-DOF, an F biosynthesis intermediate, showed reduced ABO/SGA risk. Though minimally bioactive, 11-DOF was identified as a potential biomarker for trisomy 21, and associated with GA (29). Furthermore, F/11-DOF ratio significantly increased in the SGA group, serving as a biomarker for fetal conditions (40).

Several limitations of this study should be acknowledged. First, the cohort consisted of participants with relatively high educational attainment (74.7% with a bachelor’s degree or higher), and women with diagnosed endocrine disorders (e.g., polycystic ovary syndrome or thyroid disorders) were excluded at enrollment. Therefore, the findings may be most applicable to generally healthy and relatively socioeconomically advantaged populations, which may limit generalizability to other populations. Second, although we adjusted for multiple confounders, some factors such as diet, physical activity, maternal stress, and environmental exposures were not accounted for, potentially introducing residual confounding. Third, steroid hormones were measured at a single time point in early pregnancy, which may not capture dynamic changes across gestation or reflect hormone levels during outcome-specific critical windows (e.g., mid-to-late pregnancy for PTB), potentially biasing associations toward the null. Fourth, the relatively small number of cases for individual ABO subtypes may limit statistical power. Therefore, subtype-specific analyses should be interpreted as exploratory, and null findings in underpowered subgroup analyses should be viewed with caution. Nevertheless, these analyses were conducted to explore potential heterogeneity across different ABOs, which may involve distinct biological mechanisms and clinical pathways. Fifth, some steroid hormones showed very high correlations (e.g., CORT and 21-DOF), which may introduce collinearity and affect model stability, although sensitivity analyses suggested generally consistent patterns of association. Finally, given the multiple comparisons and limited sample size for some outcomes, the findings should be considered exploratory, with emphasis placed on overall patterns of association rather than individual statistically significant results. Larger prospective studies with repeated hormone measurements and larger sample sizes are needed to validate these findings.

## Conclusions

Simultaneous measurement of multiple steroid hormones provides a more comprehensive assessment of maternal endocrine status than single-hormone analyses. In this study, higher levels of A4 and T in early pregnancy were associated with increased risks of ABO and SGA, with consistent directional patterns observed for androgen-related hormones across analytical approaches. These findings suggest a potential role of the maternal early pregnancy hormonal milieu in fetal growth and development. However, given the multiple comparisons and limited sample size for some outcomes, the results should be considered exploratory and interpreted with caution. Further studies in larger and more diverse populations are needed to confirm these findings and evaluate their potential clinical relevance.

## Data Availability

The raw data supporting the conclusions of this article will be made available by the authors, without undue reservation.
