# Supplementary material for: Steroid hormones in early pregnancy and adverse birth outcomes: a Chinese birth cohort
Source: Front Endocrinol (Lausanne). 2026 Apr 20;17:1667039. doi: 10.3389/fendo.2026.1667039 (PMC13135977; doi:10.3389/fendo.2026.1667039)
Supplement: Supplementary file 1 [file DataSheet1.docx]

**Supplementary Information**

**Steroid Hormones in Early Pregnancy and Adverse Birth Outcomes: A Chinese Birth Cohort**

**Weixiang Wu^1,2,†^, Cunwei Ji^1,2,†^, Hongyu Li^1,2^, Fuqiang Diao^1,2^, Lihong Wu^1,2^, Xiaolin Ruan^1,2^, Youwen Luo^1,2^, Mingyong Luo^1,2,*^**

^1^Department of Clinical Laboratory, Women and Children's Hospital, Southern University of Science and Technology, Guangzhou, China

^2^Department of Clinical Laboratory, Guangdong Women and Children Hospital, Guangzhou, China

^*^ Corresponding author: Department of Clinical Laboratory, Women and Children's Hospital, Southern University of Science and Technology, 521 Xingnan Road, Guangzhou 511400, China. E-mail: luo-my@163.com

^†^These authors have contributed equally to this work and share first authorship

Number of pages: 27

Number of supplementary methods: 1

Number of figures: 9

Number of tables: 15

**Methods**

**Steroid hormone analyses**

A total of 19 types steroid hormones were measured in this study, including 5 glucocorticoids (cortisol [F], cortisone [E], corticosterone [CORT], 11-deoxycortisol [11-DOF], 21-deoxycortisol [21-DOF]), 2 mineralocorticoids (aldosterone [ALD], 11-deoxycorticosterone [DOC]), 5 androgens (androstenedione [A4], T, dihydrotestosterone [DHT], dehydroepiandrosterone [DHEA], dehydroepiandrosterone sulfate [DHEAS]), 3 estrogens (estrone [E1], E2, estriol [E3]), and 4 progestogens (progesterone [P4], pregnenolone [P5], 17α-Hydroxyprogesterone [17-OHP4], 17-hydroxypregnenolone [17-OHP5]). An aliquot of 200 μL serum was firstly thawed at room temperature, and followed by the addition of 300 μL acetonitrile containing internal standards (10 ng/mL). The mixture was vortexed for 1 min and 500 μL ethyl acetate was added. After the vortex at 2000 rpm (5 min) and the following centrifugation at 12000 rpm (5 min), 600 μL of the supernatant was transferred to a 96-well plate. The extract was then concentrated using a gentle stream of N_2_, and was reconstituted with 100 μL of a methanol-water (50:50) diluent. Each sample was vortexed at 800 rpm for 5 min before analysis. The detection was conducted on an ExionLC AC liquid chromatograph coupled with a 6500 Qtrap LC-MS/MS system (AB SCIEX, Toronto, Canada). For hormones measured with positive electrospray ionization (ESI+), a Thermo Accocre PFP column (100 × 2.1 mm, 2.6 μm) was used. The mobile phase consisted of 0.1% formic acid in water (A) and 0.1% formic acid in methanol (B); the flow rate was 0.45 mL/min, and the following gradient was used: 0−3.2 min, 39% to 42% B; 3.2−7.5 min, 42% B to 90% B; 7.51−8.5 min, 100% B to 100% B; 8.51−10 min, 39% B to 39% B. For DHEAS, E1, E2, and E3 measured with ESI−, a Phenomenex Titank C18 column (100 × 2.1 mm, 3 μm) was applied. The mobile phase consisted of 0.04% ammonia in water (A) and acetonitrile (B); the flow rate was 0.4 mL/min, and the following gradient was used: 0−1.5 min, 35% B to 60% B; 1.51−3 min, 95% B to 95% B; 3.01−4.5 min, 35% B to 35% B. Details of the optimized MS/MS parameters are listed in Supplementary Tables S1 and S2.

Quality controls consisting of three grades (low, medium, and high levels) were analyzed in each batch of 40 samples to monitor precision and check instrument performance, and a blank sample was included to avoid potential contamination. If the measured values were suspected to be contaminated or deviated from the analytical values, the instrument was recalibrated, and the previous batch of samples was reanalyzed. The coefficient of variation ranged from 4.20% to 11.93%, and the limit of quantitation (LOQ) ranged from 0.003 to 0.407 ng/mL (Supplementary Table S3). A total of 17 hormones had a detection rate of 100%, E3 had a detection rate of 94.0%, and 17-OHP5 had a detection rate of 68.4%. If the sample concentration was < LOQ, a value of LOQ divided by √2 was assigned before further statistical analysis.

**
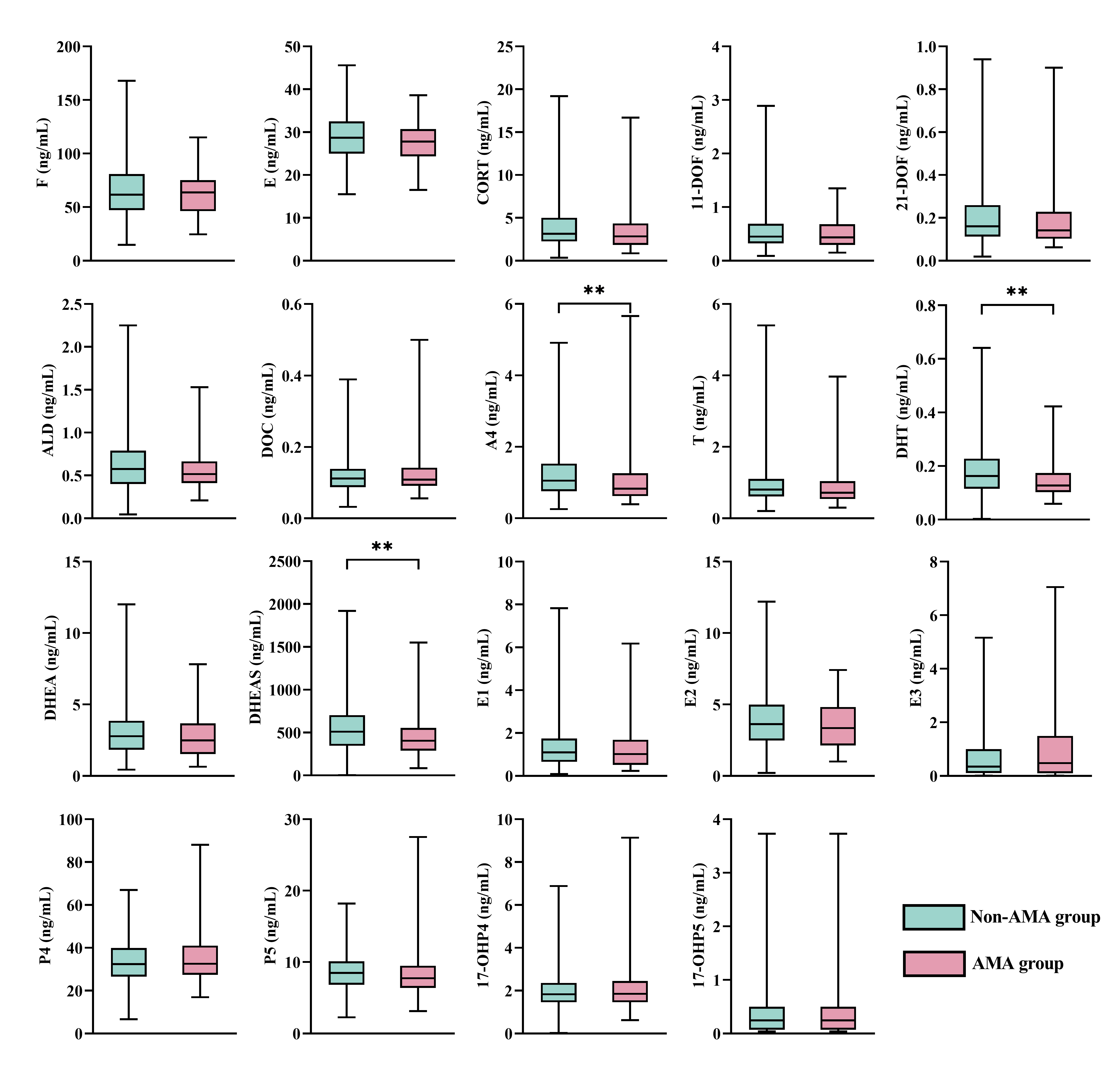
 Fig. S1** Distributions of 19 steroid hormones in pregnancy women according to AMA. Data are presented as box plots showing the median (line), interquartile range (box), and range (whiskers). The green color indicates Non-AMA group, and the red color indicates AMA group. *P*-values for differences in these groups are calculated using Mann-Whitney tests. ^*^*P* < 0.05, ^**^*P* < 0.01, ^***^*P* < 0.001.


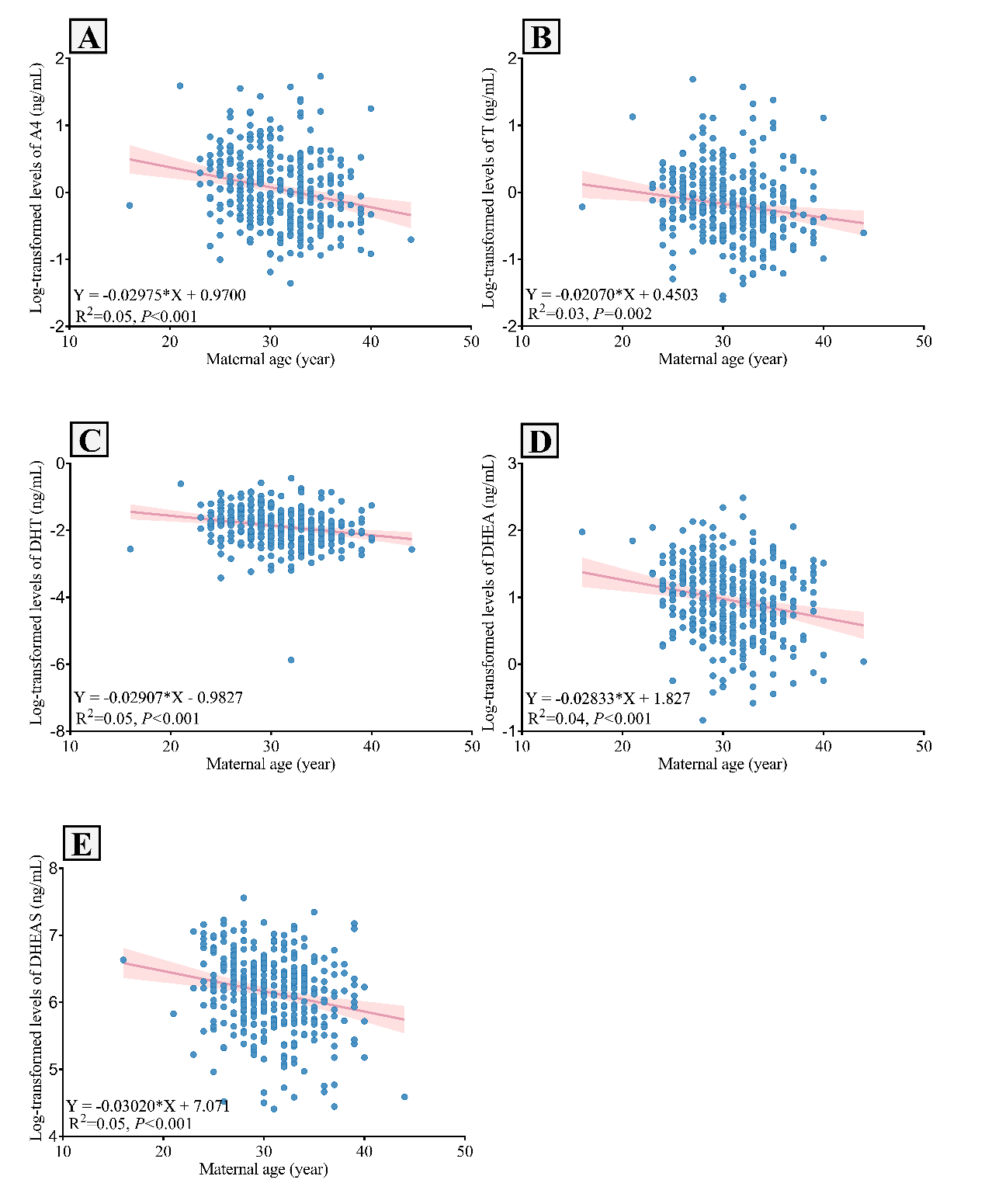


**Fig. S2** Correlations between maternal age and log-transformed levels of (A) A4, (B) T, (C) DHT, (D) DHEA, and (E) DHEAS in ng/mL. Red lines indicate the linear regression and the pink areas represent the 95% CIs.

**
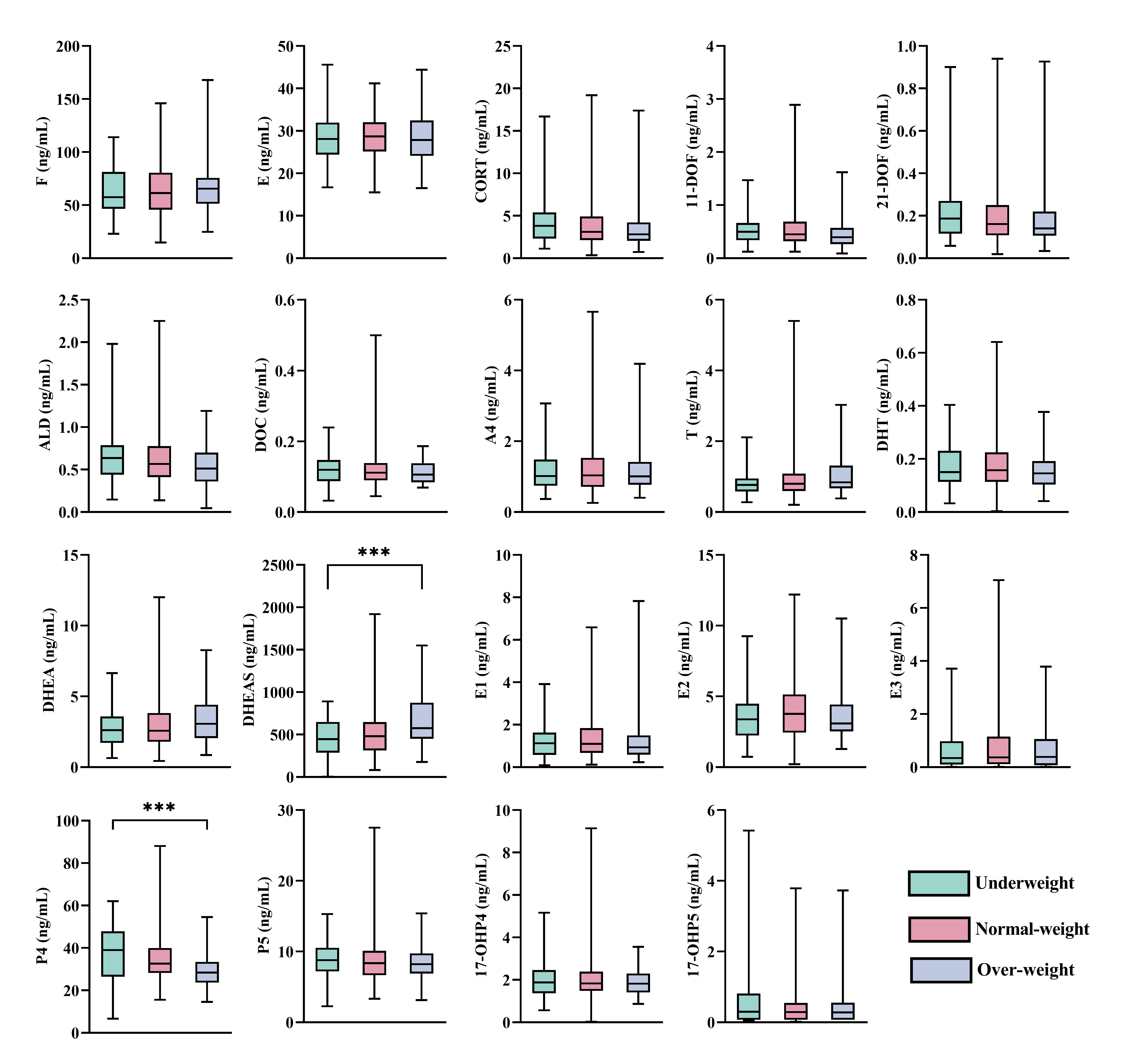
Fig. S3** Distributions of 19 steroid hormones in pregnancy women according to pre-pregnancy BMI categories. Data are presented as box plots showing the median (line), interquartile range (box), and range (whiskers). The green color indicates the underweight group, the red color indicates the normal-weight group, and the grey color indicates the over-weight group. *P*-values for differences in these groups are calculated using Kruskal-Wallis tests. ^*^*P* < 0.05, ^**^*P* < 0.01, ^***^*P* < 0.001.


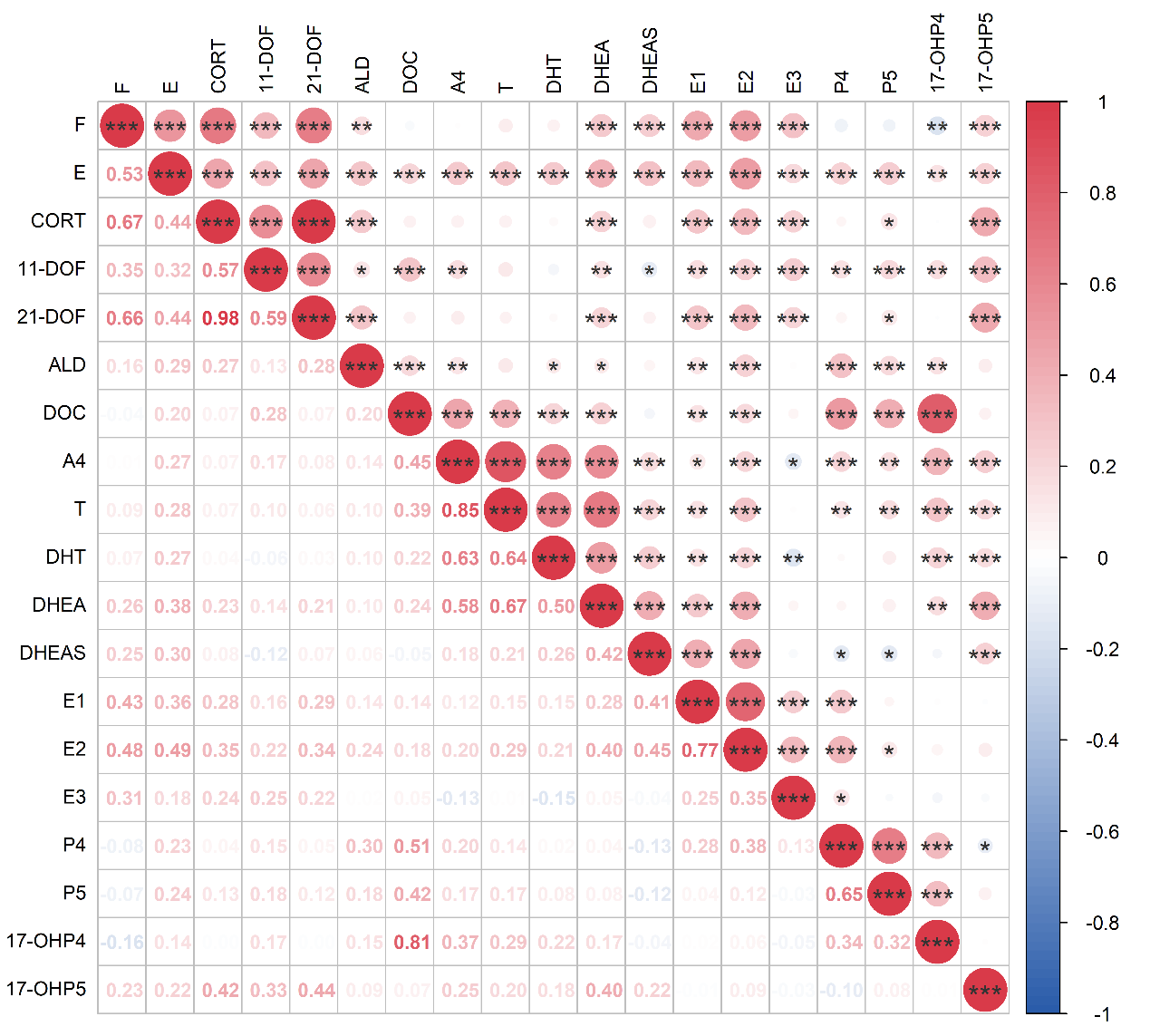


**Fig. S4** Pearson correlation matrix of 19 steroid hormones among study populations (ln-transformed). The red color representes a positive correlation, while blue color representes a negative correlation. The darker the color, the greater the correlation coefficient. ^*^*P* < 0.05, ^**^*P* < 0.01, ^***^*P* < 0.001.


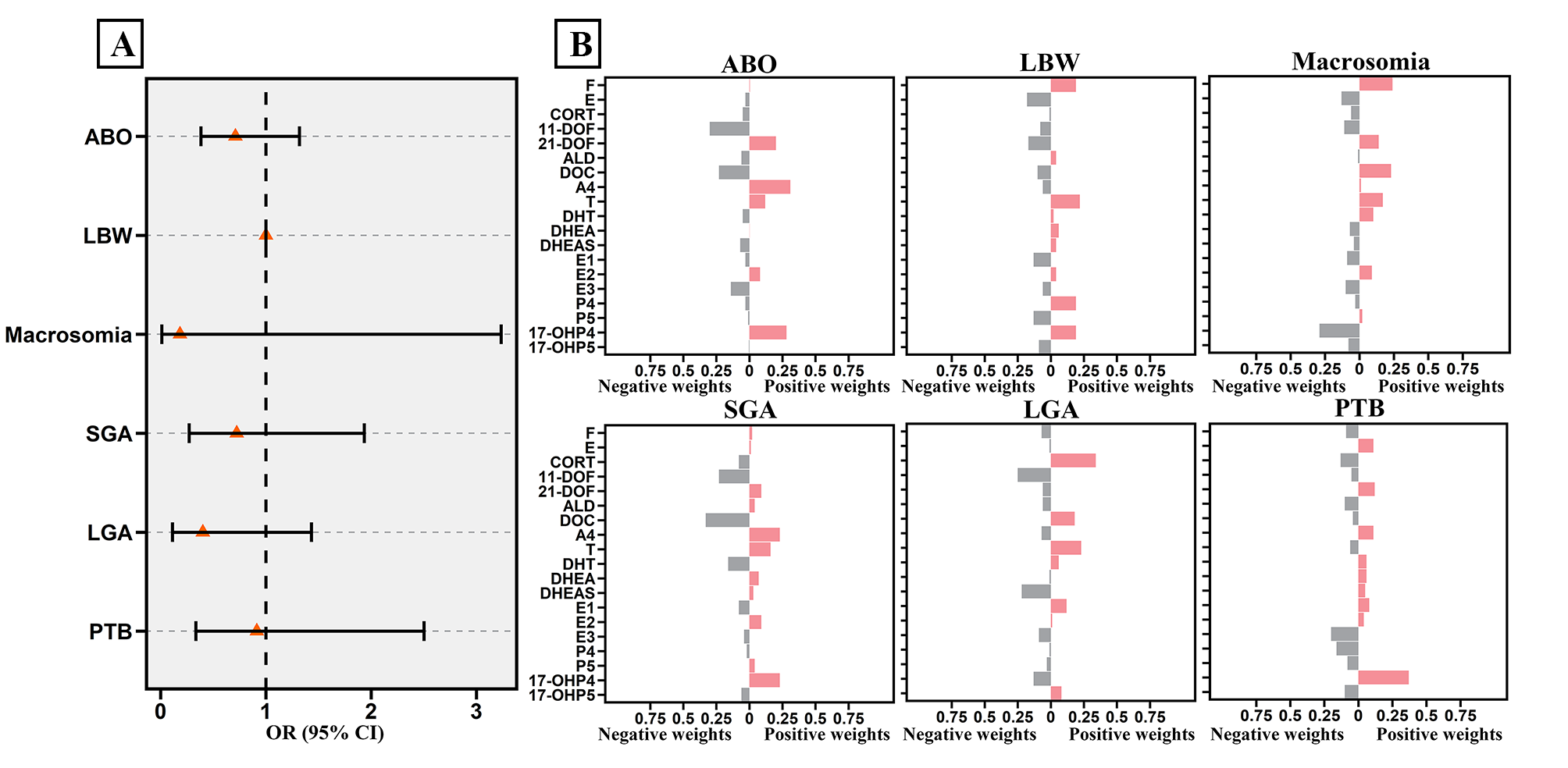


**Fig. S5** Effects of a mixture of 19 hormones on ABOs based on Qgcomp model analyses. (A) Combined effect of 19 steroid hormones on ABOs. (B) Contributions of each hormone on ABOs. The Qgcomp models were adjusted for sampling gestational week, maternal age, education level, pre-pregnancy BMI, delivery mode, parity, HDP, GDM, and infant sex for ABO, SGA, and LGA , with GA additionally adjusted for LBW and macrosomia. The red bars represent the positive weights, while the grey bars represent a negative correlation. ^*^*P* < 0.05.


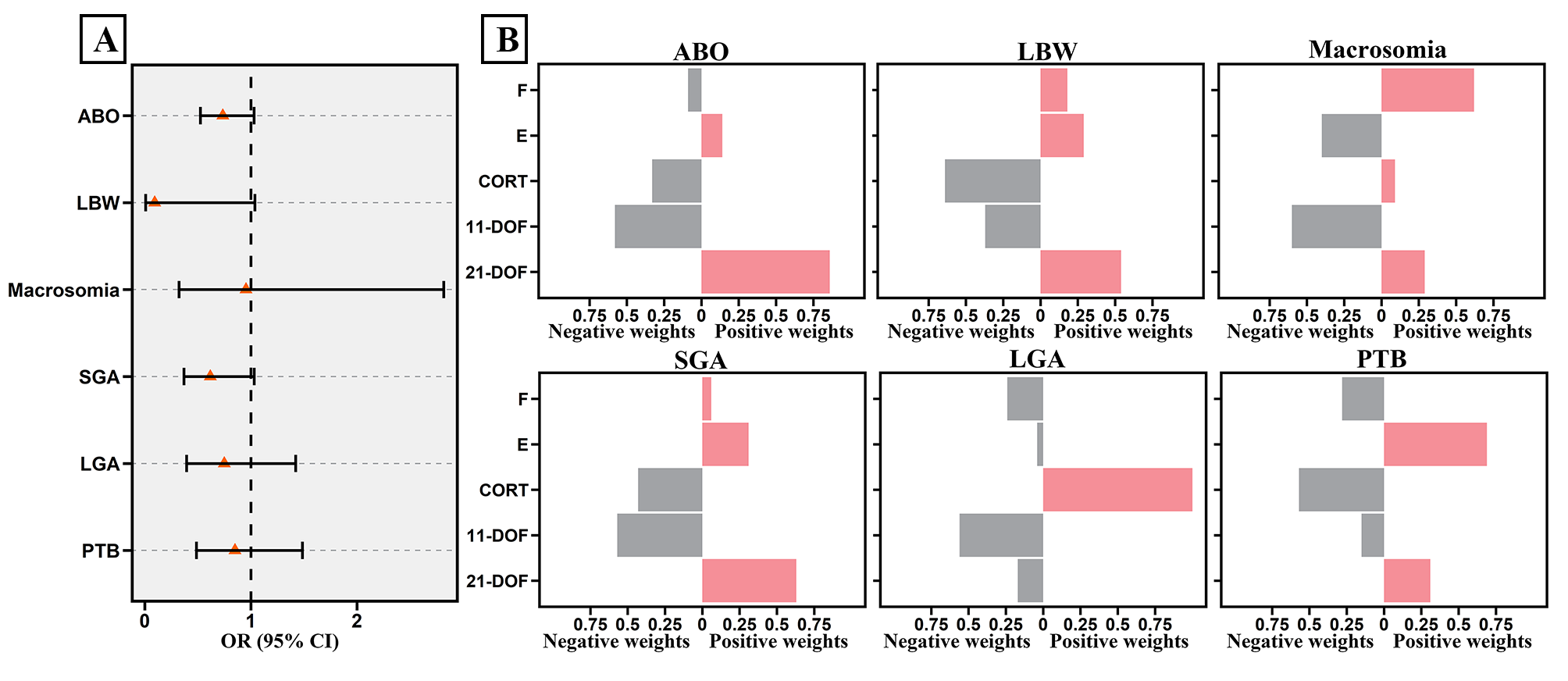


**Fig. S6** Effects of a mixture of five glucocorticoids on ABOs based on Qgcomp model analyses. (A) Combined effect of the glucocorticoid mixture on ABOs. (B) Contributions of each hormone on ABOs. The Qgcomp models were adjusted for sampling gestational week, maternal age, education level, pre-pregnancy BMI, delivery mode, parity, HDP, GDM, and infant sex for ABO, SGA, and LGA , with GA additionally adjusted for LBW and macrosomia. The red bars represent the positive weights, while the grey bars represent a negative correlation. ^*^*P* < 0.05.


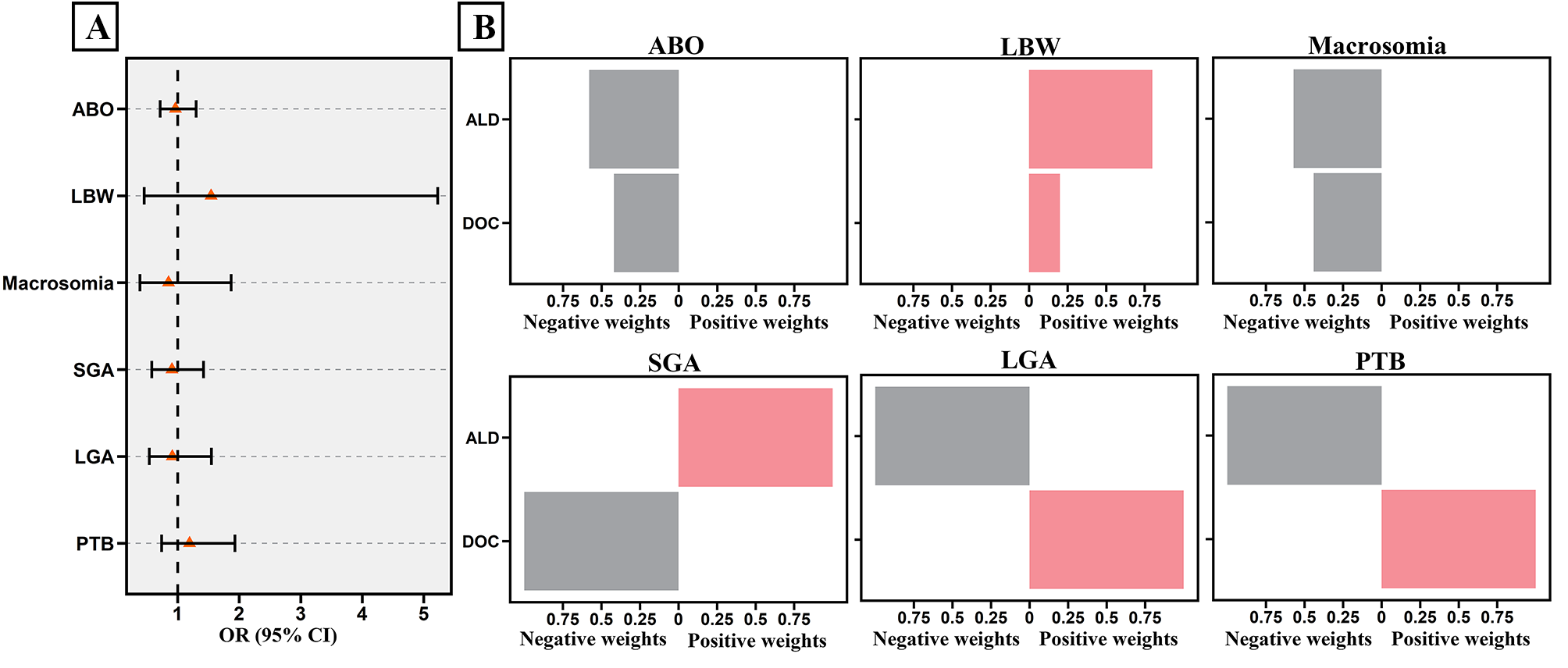


**Fig. S7** Effects of a mixture of two mineralocorticoids on ABOs based on Qgcomp model analyses. (A) Combined effect of the mineralocorticoid mixture on ABOs. (B) Contributions of each hormone on ABOs. The Qgcomp models were adjusted for sampling gestational week, maternal age, education level, pre-pregnancy BMI, delivery mode, parity, HDP, GDM, and infant sex for ABO, SGA, and LGA , with GA additionally adjusted for LBW and macrosomia. The red bars represent the positive weights, while the grey bars represent a negative correlation. ^*^*P* < 0.05.


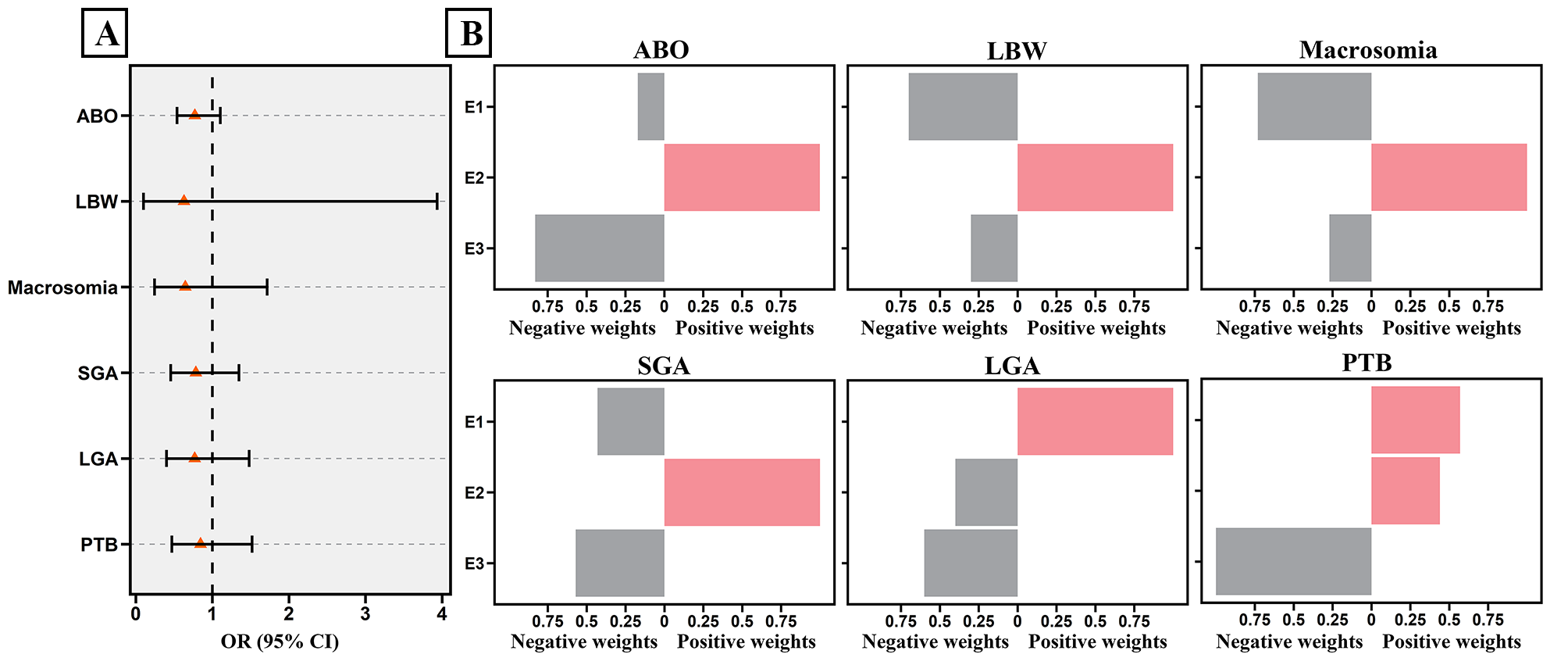


**Fig. S8** Effects of a mixture of three estrogens on ABOs based on Qgcomp model analyses. (A) Combined effect of the estrogen mixture on ABOs. (B) Contributions of each hormone on ABOs. The Qgcomp models were adjusted for sampling gestational week, maternal age, education level, pre-pregnancy BMI, delivery mode, parity, HDP, GDM, and infant sex for ABO, SGA, and LGA , with GA additionally adjusted for LBW and macrosomia. The red bars represent the positive weights, while the grey bars represent a negative correlation. ^*^*P* < 0.05.


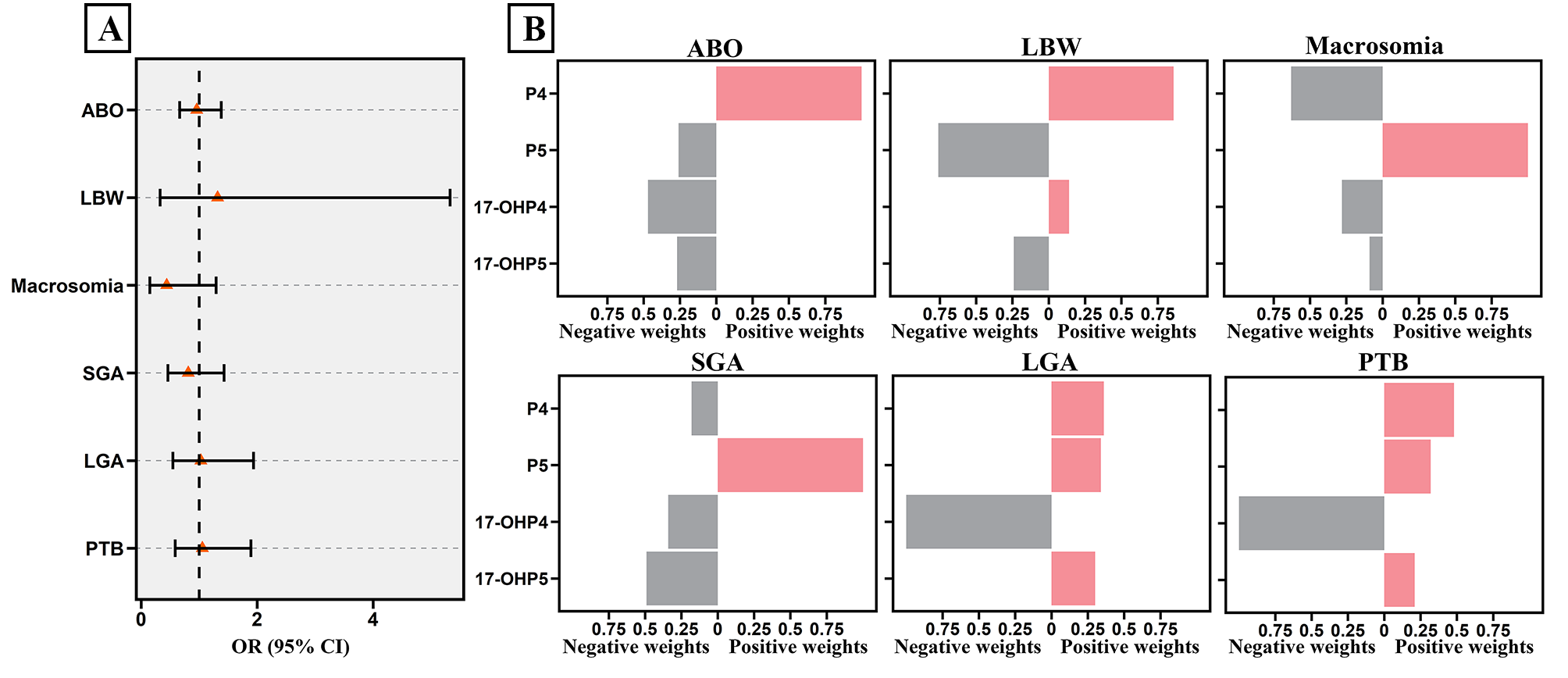


**Fig. S9** Effects of a mixture of four progestogens on ABOs based on Qgcomp model analyses. (A) Combined effect of the progestogen mixture on ABOs. (B) Contributions of each hormone on ABOs. The Qgcomp models were adjusted for sampling gestational week, maternal age, education level, pre-pregnancy BMI, delivery mode, parity, HDP, GDM, and infant sex for ABO, SGA, and LGA , with GA additionally adjusted for LBW and macrosomia. The red bars represent the positive weights, while the grey bars represent a negative correlation. ^*^*P* < 0.05.

| **Table S1** Optimized MS Parameters for the qualitative and quantitative analysis of 19 steroid hormones. | | | | | |
| --- | --- | --- | --- | --- | --- |
| Steroid Hormone | ESI | Parent Ion (m/z) | Product Ion (m/z) | Declustering Potential (eV) | Collision Energy (eV) |
| F | + | 363.3 | 121.2^a^ | 100 | 25 |
|  | + |  | 155.1 | 80 | 25 |
| E | + | 361.3 | 163.1^a^ | 90 | 32 |
|  | + |  | 121.3 | 90 | 40 |
| CORT | + | 347.2 | 121.1^a^ | 65 | 30 |
|  | + |  | 329.2 | 65 | 21 |
| 11-DOF | + | 347.1 | 97.1^a^ | 100 | 30 |
| 21-DOF | + | 347.1 | 311.2^a^ | 100 | 21 |
| ALD | + | 361.3 | 343.3^a^ | 60 | 24 |
|  | + |  | 325.1 | 60 | 24 |
| DOC | + | 331.2 | 97.2^a^ | 80 | 27 |
|  | + |  | 295 | 80 | 27 |
| A4 | + | 287.1 | 97.1^a^ | 85 | 30 |
|  | + |  | 109.2 | 85 | 31 |
| T | + | 289.2 | 97.2^a^ | 90 | 28 |
|  | + |  | 109 | 90 | 33 |
| DHT | + | 291.3 | 255.2^a^ | 50 | 23 |
|  | + |  | 273.3 | 50 | 20 |
| DHEA | + | 289.2 | 271^a^ | 70 | 20 |
|  | + |  | 253 | 70 | 20 |
| DHEAS | − | 367.1 | 97^a^ | −120 | −30 |
|  | − |  | 80 | −120 | −80 |
| E1 | − | 269.2 | 145^a^ | −80 | −50 |
|  | − |  | 159 | −80 | −45 |
| E2 | − | 271.4 | 145^a^ | −150 | −50 |
|  | − |  | 183 | −150 | −50 |
| E3 | − | 287.2 | 145.1^a^ | −80 | −50 |
|  | − |  | 171.2 | −80 | −51 |
| P4 | + | 315.2 | 97.1^a^ | 50 | 28 |
|  | + |  | 109.1 | 50 | 30 |
| P5 | + | 317.2 | 159.2^a^ | 45 | 32 |
|  | + |  | 299.1 | 45 | 17 |
| 17-OHP4 | + | 331.2 | 97.2^a^ | 80 | 30 |
|  | + |  | 109.2 | 80 | 33 |
| 17-OHP5 | + | 315.2 | 297.2^a^ | 60 | 20 |
|  | + |  | 133.2 | 60 | 32 |
| Abbreviations: F, cortisol; E, cortisone; CORT, corticosterone; 11-DOF, 11-deoxycortisol; 21-DOF, 21-deoxycortisol; ALD, aldosterone; DOC, 11-deoxycorticosterone; A4, androstenedione; T, testosterone; DHT, dihydrotestosterone; DHEA, dehydroepiandrosterone; DHEAS, dehydroepiandrosterone sulfate; E1, estrone; E2, estradiol; E3, estriol; P4, progesterone; P5, pregnenolone; 17-OHP4, 17α-hydroxyprogesterone; 17-OHP5, 17-hydroxypregnenolone. ^a^ quantitative ion; | | | | | |

| **Table S2** Optimized MS parameters for the quantitative analysis of internal standards. | | | | | |
| --- | --- | --- | --- | --- | --- |
| Steroid Hormone | ESI | Parent Ion (m/z) | Product Ion (m/z) | Declustering Potential (eV) | Collision Energy (eV) |
| E*-d*7 | + | 368.4 | 168.1 | 40 | 32 |
| CORT*-d*8 | + | 355.2 | 337.1 | 65 | 22 |
| 21*-*DOF*-d*8 | + | 355.3 | 125.1 | 100 | 30 |
| A4*-d*7 | + | 294.3 | 100.2 | 40 | 30 |
| T*-d*5 | + | 294.1 | 100.3 | 40 | 30 |
| DHT*-d*4 | + | 295.1 | 259.2 | 40 | 24 |
| DHEAS*-d*6 | − | 373.1 | 98 | −150 | −38 |
| E1*-d*4 | − | 273.3 | 147 | −80 | −49 |
| E2*-d*5 | − | 276.4 | 147 | −150 | −50 |
| P4*-d*9 | + | 324.1 | 100.3 | 55 | 30 |
| P5*-d*4 | + | 321.2 | 303.4 | 45 | 20 |
| 17-OHP4*-d*8 | + | 339.1 | 100.1 | 80 | 35 |
| 17-OHP5*-d*3 | + | 336.2 | 300.2 | 100 | 15 |

| **Table S3** Recovery, coefficient of variation, limit of quantitation, and detection rate in our study. | | | | | | |
| --- | --- | --- | --- | --- | --- | --- |
| Steroid Hormone | %Recovery (mean ± SD) | | | CV (%) | LOQ (ng/mL) | >LOQ % |
|  | QC (low) | QC (medium) | QC (high) |  |  |  |
| F | 108.27 ± 6.43 | 110.85 ± 10.53 | 92.08 ± 5.45 | 9.01% | 0.025 | 100% |
| E | 100.47 ± 5.90 | 103.33 ± 5.21 | 99.25 ± 2.77 | 4.78% | 0.011 | 100% |
| CORT | 102.73 ± 5.79 | 99.97 ± 4.58 | 100.67 ± 7.76 | 4.35% | 0.033 | 100% |
| 11-DOF | 100.53 ± 7.79 | 103.63 ± 4.59 | 103.09 ± 13.85 | 4.20% | 0.010 | 100% |
| 21-DOF | 109.40 ± 8.63 | 101.00 ± 5.26 | 99.75 ± 6.80 | 4.94% | 0.003 | 100% |
| ALD | 94.60 ± 7.58 | 105.91 ± 7.35 | 99.89 ± 2.55 | 6.58% | 0.050 | 100% |
| DOC | 103.57 ± 5.29 | 100.93 ± 5.31 | 99.25 ± 4.98 | 4.99% | 0.004 | 100% |
| A4 | 101.80 ± 4.79 | 101.87 ± 5.80 | 97.31 ± 6.33 | 5.40% | 0.011 | 100% |
| T | 97.87 ± 5.84 | 101.03 ± 6.38 | 100.77 ± 6.33 | 5.99% | 0.008 | 100% |
| DHT | 97.20 ± 14.36 | 103.43 ± 6.08 | 103.33 ± 8.76 | 5.58% | 0.016 | 100% |
| DHEA | 104.1 ± 5.48 | 106.13 ± 8.66 | 101.57 ± 10.32 | 7.74% | 0.407 | 100% |
| DHEAS | 110.15 ± 3.68 | 104.63 ± 5.59 | 96.53 ± 5.69 | 5.07% | 0.137 | 100% |
| E1 | 97.77 ± 11.52 | 100.20 ± 11.64 | 94.91 ± 7.46 | 7.86% | 0.018 | 100% |
| E2 | 102.53 ± 9.39 | 108.33 ± 9.03 | 98.17 ± 6.31 | 7.91% | 0.010 | 100% |
| E3 | 112.10 ± 14.10 | 98.93 ± 17.36 | 97.43 ± 19.07 | 11.93% | 0.010 | 94% |
| P4 | 106.07 ± 7.59 | 103.93 ± 8.49 | 102.07 ± 6.03 | 7.75% | 0.012 | 100% |
| P5 | 100.77 ± 6.66 | 97.47 ± 8.83 | 102.75 ± 7.05 | 8.60% | 0.082 | 100% |
| 17-OHP4 | 99.75 ± 5.07 | 100.87 ± 5.29 | 100.13 ± 3.59 | 4.98% | 0.083 | 100% |
| 17-OHP5 | 109.40 ± 13.31 | 98.80 ± 8.23 | 104.40 ± 7.54 | 7.90% | 0.100 | 68% |
| Note: QC, quality control; CV, coefficient of variation; LOQ, limit of quantitation; >LOQ %, detection rate. | | | | | | |

| **Table S4** Model specifications and covariate adjustment for regression and Qgcomp analyses | | | | |
| --- | --- | --- | --- | --- |
| Model | Outcome | Exposure | GA status | Covariates |
| Linear regression | BW, BL, HC | Individual hormones | Including GA | Sampling gestational week, maternal age, education level, pre-pregnancy BMI, delivery mode, parity, HDP, GDM, infant sex, and GA |
| Linear regression | GA | Individual hormones | Excluding GA | Sampling gestational week, maternal age, education level, pre-pregnancy BMI, delivery mode, parity, HDP, GDM, and infant sex |
| Logistic regression | LBW, macrosomia | Individual hormones/  hormone mixtures | Including GA | Sampling gestational week, maternal age, education level, pre-pregnancy BMI, delivery mode, parity, HDP, GDM, infant sex, and GA |
| Logistic regression | ABO, SGA, LGA, PTB | Individual hormones/  hormone mixtures | Excluding GA | Sampling gestational week, maternal age, education level, pre-pregnancy BMI, delivery mode, parity, HDP, GDM, and infant sex |
| Qgcomp | LBW, macrosomia | Total hormone mixtures | Including GA | Sampling gestational week, maternal age, education level, pre-pregnancy BMI, delivery mode, parity, HDP, GDM, infant sex, and GA |
| Qgcomp | ABO, SGA, LGA, PTB | Total hormone mixtures | Excluding GA | Sampling gestational week, maternal age, education level, pre-pregnancy BMI, delivery mode, parity, HDP, GDM, and infant sex |
| Qgcomp | LBW, macrosomia | Class-specific hormone mixture | Including GA | Sampling gestational week, maternal age, education level, pre-pregnancy BMI, delivery mode, parity, HDP, GDM, infant sex, and GA |
| Qgcomp | ABO, SGA, LGA, PTB | Class-specific hormone mixture | Excluding GA | Sampling gestational week, maternal age, education level, pre-pregnancy BMI, delivery mode, parity, HDP, GDM, and infant sex |
| Note: GA was included as a covariate for outcomes not defined by gestational age and excluded for GA-dependent outcomes (SGA, LGA, PTB) and for GA itself to avoid overadjustment. For ABO, GA was not included due to overlap with GA-related components. QgComp models used the same covariates as the corresponding regression models. | | | | |

**Table S5** Characteristics of 364 pregnant women and newborns.

| Characteristics | Mean ± SD or n (%) |
| --- | --- |
| Maternal age (year) | 30.7 ± 4.0 |
| Pre-pregnancy BMI (kg/m^2^) | 21.2 ± 3.0 |
| Pre-pregnancy BMI categories |  |
| Underweight (< 18.5 kg/m^2^) | 50 (13.7) |
| Normal-weight (18.5-23.9 kg/m^2^) | 254 (69.8) |
| Overweight (>23.9 kg/m^2^) | 60 (16.5) |
| Level of education |  |
| Less than bachelor degree | 92 (25.3) |
| Bachelor degree or above | 272 (74.7) |
| Mode of delivery |  |
| Vaginal delivery | 228 (62.6) |
| Cesarean section | 136 (37.4) |
| Nulliparous | 191 (52.5) |
| Hypertension during pregnancy | 9 (2.5) |
| Gestational diabetes | 62 (17.0) |
| Gestational age at sampling (week) | 14.2 ± 1.5 |
| Male infant | 199 (54.7) |
| Birth parameter |  |
| GA (week) | 39.1 ± 1.3 |
| BW (g) | 3191.7 ± 407.1 |
| BL (cm) | 49.4 ± 1.7 |
| HC (cm) | 33.6 ± 1.4 |
| ABO |  |
| LBW | 14 (3.9) |
| SGA | 32 (8.7) |
| Macrosomia | 11 (3.0) |
| LGA | 24 (6.5) |
| PTB | 28 (7.7) |
| Abbreviations: SD, standard deviation; BMI, body mass index; GA, Gestational age at delivery; BW, birth weight; BL, birth length; HC, head circumference; SGA, small-for-gestational age; LGA, large-for-gestational age; PTB, preterm birth. Continuous variables are presented as mean ± SD, and categorical variables are presented as n (%). | |

| **Table S6** Distribution characteristics of 19 steroid hormones according to ABO group. | | | | | | |
| --- | --- | --- | --- | --- | --- | --- |
| Steroid hormone | Subgroup | GM | Mean | Median (IQR) | Range | *P*-value |
| F | Non-ABO | 60.6 | 65.7 | 62.8 (47.2−79.8) | 14.7−158.0 | 0.531 |
|  | ABO | 57.8 | 63.4 | 60.4 (45.4−79.0) | 14.7−130.0 |  |
| E | Non-ABO | 28.1 | 28.7 | 28.6 (24.7−32.1) | 15.5−45.6 | 0.504 |
|  | ABO | 27.7 | 28.2 | 27.7 (25.0−31.9) | 17.4−40.4 |  |
| CORT | Non-ABO | 3.35 | 4.15 | 3.13 (2.23−5.07) | 0.36−19.2 | 0.182 |
|  | ABO | 2.94 | 3.65 | 2.98 (1.68−4.32) | 0.77−15.6 |  |
| 11-DOF | Non-ABO | 0.47 | 0.57 | 0.47 (0.32−0.71) | 0.09−2.89 | 0.020^*^ |
|  | ABO | 0.41 | 0.47 | 0.38 (0.27−0.58) | 0.14−2.31 |  |
| 21-DOF | Non-ABO | 0.17 | 0.21 | 0.16 (0.11−0.26) | 0.02−0.94 | 0.148 |
|  | ABO | 0.15 | 0.19 | 0.15 (0.09−0.23) | 0.05−0.87 |  |
| ALD | Non-ABO | 0.55 | 0.61 | 0.56 (0.41−0.76) | 0.05−1.98 | 0.740 |
|  | ABO | 0.54 | 0.63 | 0.57 (0.37−0.78) | 0.15−2.25 |  |
| DOC | Non-ABO | 0.11 | 0.12 | 0.11 (0.09−0.14) | 0.03−0.50 | 0.889 |
|  | ABO | 0.11 | 0.12 | 0.11 (0.09−0.14) | 0.07−0.29 |  |
| A4 | Non-ABO | 1.01 | 1.17 | 0.99 (0.71−1.40) | 0.26−5.66 | 0.005^**^ |
|  | ABO | 1.22 | 1.43 | 1.34 (0.76−1.82) | 0.43−4.84 |  |
| T | Non-ABO | 0.80 | 0.91 | 0.78 (0.59−1.04) | 0.20−5.40 | 0.014^*^ |
|  | ABO | 0.95 | 1.13 | 0.89 (0.65−1.36) | 0.30−4.83 |  |
| DHT | Non-ABO | 0.15 | 0.17 | 0.15 (0.11−0.21) | 0.02−0.56 | 0.068 |
|  | ABO | 0.17 | 0.19 | 0.17 (0.12−0.23) | 0.06−0.64 |  |
| DHEA | Non-ABO | 2.57 | 2.95 | 2.55 (1.79−3.73) | 0.43−12.0 | 0.174 |
|  | ABO | 2.74 | 3.23 | 3.01 (2.00−4.15) | 0.56−9.07 |  |
| DHEAS | Non-ABO | 452.5 | 529.3 | 484.0 (339.8−675.5) | 82.1−1420.0 | 0.599 |
|  | ABO | 482.1 | 560.3 | 521.0 (307.8−704.8) | 85.2−1350.0 |  |
| E1 | Non-ABO | 1.04 | 1.38 | 1.09 (0.63−1.77) | 0.09−7.83 | 0.813 |
|  | ABO | 1.03 | 1.23 | 1.12 (0.62−1.58) | 0.20−3.69 |  |
| E2 | Non-ABO | 3.41 | 3.90 | 3.60 (2.43−4.98) | 0.21−12.2 | 0.871 |
|  | ABO | 3.42 | 3.82 | 3.53 (2.54−4.87) | 1.01−9.31 |  |
| E3 | Non-ABO | 0.33 | 0.81 | 0.40 (0.12−1.18) | <LOQ−7.05 | 0.026^*^ |
|  | ABO | 0.20 | 0.60 | 0.29 (0.06−0.79) | <LOQ−3.52 |  |
| P4 | Non-ABO | 32.6 | 34.2 | 32.6 (26.7−40.8) | 6.66−88.1 | 0.810 |
|  | ABO | 32.8 | 34.1 | 31.7 (26.6−38.8) | 18.7−67.0 |  |
| P5 | Non-ABO | 8.22 | 8.63 | 8.42 (6.83−10.1) | 2.25−27.5 | 0.866 |
|  | ABO | 8.25 | 8.60 | 8.24 (6.58−10.1) | 3.94−15.8 |  |
| 17-OHP4 | Non-ABO | 1.83 | 2.02 | 1.83 (1.46−2.36) | 0.08−9.14 | 0.463 |
|  | ABO | 1.91 | 2.03 | 1.87 (1.53−2.46) | 0.95−5.53 |  |
| 17-OHP5 | Non-ABO | 0.26 | 0.50 | 0.29 (<LOQ−0.57) | <LOQ−5.42 | 0.736 |
|  | ABO | 0.24 | 0.40 | 0.28 (<LOQ−0.51) | <LOQ−1.78 |  |
| ^*^*P* < 0.05, ^**^*P* < 0.01. | | | | | | |

| **Table S7** Multiple regression models on the association between steroid hormones (per 1-SD increase) and LBW, macrosomia, and PTB risk. | | | | | | | | |
| --- | --- | --- | --- | --- | --- | --- | --- | --- |
| Steroid Hormones  (SD, ng/mL) | LBW | |  | Macrosomia | |  | PTB | |
|  | OR (95% CI) | *P*-value |  | OR (95% CI) | *P*-value |  | OR (95% CI) | *P*-value |
| F (25.78) | 0.35 (0.09, 1.39) | 0.136 |  | 1.91 (0.88, 4.14) | 0.101 |  | 0.90 (0.56, 1.42) | 0.639 |
| E (5.62) | 0.54 (0.16, 1.82) | 0.319 |  | 1.11 (0.56, 2.21) | 0.771 |  | 0.93 (0.61, 1.43) | 0.740 |
| CORT (3.09) | 0.08 (0.00, 1.51) | 0.092 |  | 1.40 (0.84, 2.34) | 0.191 |  | 0.66 (0.36, 1.22) | 0.187 |
| 11-DOF (0.36) | 0.25 (0.04, 1.47) | 0.125 |  | 0.48 (0.16, 1.46) | 0.199 |  | 0.81 (0.49, 1.32) | 0.395 |
| 21-DOF (0.16) | 0.08 (0.01, 1.34) | 0.08 |  | 1.47 (0.89, 2.43) | 0.137 |  | 0.66 (0.35, 1.21) | 0.180 |
| ALD (0.3) | 1.13 (0.34, 3.71) | 0.847 |  | 1.23 (0.60, 2.51) | 0.574 |  | 1.11 (0.71, 1.74) | 0.656 |
| DOC (0.05) | 0.83 (0.40, 1.72) | 0.615 |  | 0.83 (0.34, 2.03) | 0.685 |  | 1.05 (0.75, 1.48) | 0.765 |
| A4 (0.78) | 1.07 (0.52, 2.21) | 0.857 |  | 1.34 (0.71, 2.50) | 0.365 |  | 1.27 (0.89, 1.83) | 0.189 |
| T (0.62) | 1.32 (0.75, 2.30) | 0.335 |  | 1.48 (0.77, 2.85) | 0.236 |  | 1.19 (0.81, 1.72) | 0.375 |
| DHT (0.09) | 1.30 (0.57, 2.99) | 0.529 |  | 1.40 (0.67, 2.90) | 0.372 |  | 1.34 (0.91, 1.99) | 0.143 |
| DHEA (1.67) | 1.58 (0.68, 3.63) | 0.285 |  | 1.29 (0.70, 2.35) | 0.412 |  | 1.14 (0.78, 1.68) | 0.490 |
| DHEAS (284.49) | 2.10 (0.60, 7.31) | 0.245 |  | 1.09 (0.54, 2.21) | 0.808 |  | 1.20 (0.79, 1.81) | 0.396 |
| E1 (1.07) | 0.52 (0.13, 2.17) | 0.371 |  | 0.59 (0.17, 2.02) | 0.399 |  | 1.05 (0.66, 1.69) | 0.830 |
| E2 (1.95) | 1.64 (0.54, 4.94) | 0.381 |  | 0.80 (0.33, 1.96) | 0.631 |  | 1.07 (0.66, 1.74) | 0.771 |
| E3 (0.98) | 0.93 (0.30, 2.87) | 0.901 |  | 0.31 (0.07, 1.43) | 0.132 |  | 0.83 (0.47, 1.46) | 0.516 |
| P4 (10.59) | 1.21 (0.60, 2.43) | 0.59 |  | 0.41 (0.15, 1.10) | 0.077 |  | 1.09 (0.73, 1.63) | 0.669 |
| P5 (2.75) | 0.89 (0.47, 1.71) | 0.736 |  | 0.75 (0.38, 1.45) | 0.39 |  | 0.85 (0.56, 1.29) | 0.446 |
| 17-OHP4 (0.95) | 0.75 (0.30, 1.88) | 0.545 |  | 0.86 (0.36, 2.06) | 0.74 |  | 1.11 (0.80, 1.55) | 0.541 |
| 17-OHP5 (0.64) | 0.29 (0.03, 2.45) | 0.253 |  | 1.15 (0.64, 2.07) | 0.631 |  | 0.62 (0.30, 1.29) | 0.202 |
| Note: Hormone concentrations were standardized using z-score transformation. Models were adjusted for sampling gestational week, maternal age, education level, pre-pregnancy BMI, delivery mode, parity, HDP, GDM, and infant sex for ABO, SGA, LGA, and PTB, with GA additionally adjusted for LBW and macrosomia. ^*^*P* < 0.05. | | | | | | | | |

| **Table S8** FDR-adjusted p-values for associations between maternal steroid hormones and birth outcomes in the main analysis. | | | | | | | | | | |
| --- | --- | --- | --- | --- | --- | --- | --- | --- | --- | --- |
| Hormone | *P*-value (FDR-adjusted *P*-values） | | | | | | | | | |
|  | BW | BL | HC | GA | ABO | SGA | LGA | LBW | Macrosomia | PTB |
| F | 0.061 (0.447) | 0.032 (0.608) | 0.017 (0.323) | 0.339 (0.719) | 0.356 (0.676) | 0.021 (0.200) | 0.850 (0.980) | 0.136 (0.646) | 0.101 (0.630) | 0.639 (0.814) |
| E | 0.094 (0.447) | 0.239 (0.821) | 0.483 (0.771) | 0.568 (0.719) | 0.558 (0.757) | 0.533 (0.633) | 0.776 (0.980) | 0.319 (0.658) | 0.771 (0.808) | 0.740 (0.814) |
| CORT | 0.060 (0.447) | 0.149 (0.821) | 0.099 (0.499) | 0.566 (0.719) | 0.246 (0.584) | 0.158 (0.439) | 0.704 (0.980) | 0.092 (0.646) | 0.191 (0.630) | 0.187 (0.768) |
| 11-DOF | 0.336 (0.673) | 0.951 (0.991) | 0.940 (0.940) | 0.442 (0.719) | 0.037 (0.176) | 0.032 (0.200) | 0.091 (0.470) | 0.125 (0.646) | 0.199 (0.630) | 0.395 (0.814) |
| 21-DOF | 0.071 (0.447) | 0.150 (0.821) | 0.105 (0.499) | 0.503 (0.719) | 0.283 (0.597) | 0.197 (0.439) | 0.677 (0.980) | 0.080 (0.646) | 0.137 (0.630) | 0.180 (0.768) |
| ALD | 0.879 (0.963) | 0.541 (0.844) | 0.276 (0.746) | 0.819 (0.865) | 0.414 (0.715) | 0.276 (0.477) | 0.910 (0.980) | 0.847 (0.901) | 0.574 (0.799) | 0.656 (0.814) |
| DOC | 0.469 (0.685) | 0.384 (0.821) | 0.541 (0.771) | 0.093 (0.442) | 0.735 (0.875) | 0.441 (0.559) | 0.671 (0.980) | 0.615 (0.779) | 0.685 (0.808) | 0.765 (0.814) |
| A4 | 0.816 (0.963) | 0.622 (0.844) | 0.356 (0.752) | 0.542 (0.719) | 0.012 (0.114) | 0.042 (0.200) | 0.074 (0.470) | 0.857 (0.901) | 0.365 (0.652) | 0.189 (0.768) |
| T | 0.706 (0.958) | 0.582 (0.844) | 0.822 (0.919) | 0.355 (0.719) | 0.009 (0.114) | 0.013 (0.200) | 0.099 (0.470) | 0.335 (0.658) | 0.236 (0.641) | 0.375 (0.814) |
| DHT | 0.828 (0.963) | 0.910 (0.991) | 0.306 (0.746) | 0.084 (0.442) | 0.027 (0.171) | 0.168 (0.439) | 0.053 (0.470) | 0.529 (0.779) | 0.372 (0.652) | 0.143 (0.768) |
| DHEA | 0.271 (0.673) | 0.432 (0.821) | 0.878 (0.927) | 0.312 (0.719) | 0.154 (0.488) | 0.208 (0.439) | 0.341 (0.980) | 0.285 (0.658) | 0.412 (0.652) | 0.490 (0.814) |
| DHEAS | 0.396 (0.673) | 0.765 (0.908) | 0.099 (0.499) | 0.372 (0.719) | 0.477 (0.755) | 0.153 (0.439) | 0.558 (0.980) | 0.245 (0.658) | 0.808 (0.808) | 0.396 (0.814) |
| E1 | 0.425 (0.673) | 0.409 (0.821) | 0.705 (0.837) | 0.010 (0.190) | 0.539 (0.757) | 0.345 (0.504) | 0.980 (0.980) | 0.371 (0.658) | 0.399 (0.652) | 0.830 (0.830) |
| E2 | 0.261 (0.673) | 0.402 (0.821) | 0.493 (0.771) | 0.384 (0.719) | 0.846 (0.893) | 0.621 (0.694) | 0.764 (0.980) | 0.381 (0.658) | 0.631 (0.799) | 0.771 (0.814) |
| E3 | 0.371 (0.673) | 0.410 (0.821) | 0.550 (0.771) | 0.769 (0.859) | 0.109 (0.414) | 0.340 (0.504) | 0.167 (0.635) | 0.901 (0.901) | 0.132 (0.630) | 0.516 (0.814) |
| P4 | 0.912 (0.963) | 0.991 (0.991) | 0.305 (0.746) | 0.735 (0.859) | 0.737 (0.875) | 0.759 (0.801) | 0.941 (0.980) | 0.590 (0.779) | 0.077 (0.630) | 0.669 (0.814) |
| P5 | 0.996 (0.996) | 0.682 (0.864) | 0.618 (0.783) | 0.945 (0.945) | 0.796 (0.890) | 0.869 (0.869) | 0.911 (0.980) | 0.736 (0.874) | 0.390 (0.652) | 0.446 (0.814) |
| 17-OHP4 | 0.418 (0.673) | 0.420 (0.821) | 0.568 (0.771) | 0.082 (0.442) | 0.975 (0.975) | 0.387 (0.525) | 0.447 (0.980) | 0.545 (0.779) | 0.740 (0.808) | 0.541 (0.814) |
| 17-OHP5 | 0.118 (0.448) | 0.542 (0.844) | 0.314 (0.746) | 0.422 (0.719) | 0.211 (0.573) | 0.232 (0.441) | 0.929 (0.980) | 0.253 (0.658) | 0.631 (0.799) | 0.202 (0.768) |
| **Note:** Values are presented as raw *P*-values (FDR-adjusted *P*-values). FDR correction was performed using the Benjamini-Hochberg method within each outcome across all hormones. No associations remained statistically significant after FDR correction. | | | | | | | | | | |

| **Table S9** Sensitivity analyses of associations between steroid hormones (ln-transformed) and birth parameters. | | | | | | | | | | | |
| --- | --- | --- | --- | --- | --- | --- | --- | --- | --- | --- | --- |
| Steroid Hormones | Birth weight (g) | |  | Birth length (cm) | |  | Birth HC (cm) | |  | GA (week) | |
|  | β (95% CI) | *P*-value |  | β (95% CI) | *P*-value |  | β (95% CI) | *P*-value |  | β (95% CI) | *P*-value |
| F | 84.90 (−1.19, 170.99) | 0.054 |  | **0.38 (0.00, 0.76)** | **0.049*** |  | **0.34 (0.01, 0.66)** | **0.042^*^** |  | −0.17 (−0.52, 0.17) | 0.32 |
| E | 135.37 (−38.78, 309.52) | 0.129 |  | 0.43 (−0.33, 1.19) | 0.27 |  | 0.26 (−0.40, 0.91) | 0.443 |  | −0.22 (−0.91, 0.47) | 0.534 |
| CORT | 46.93 (−8.99, 102.84) | 0.101 |  | 0.24 (−0.01, 0.48) | 0.058 |  | 0.19 (−0.02, 0.40) | 0.072 |  | 0.12 (−0.10, 0.34) | 0.284 |
| 11-DOF | 28.29 (−34.29, 90.87) | 0.376 |  | 0.02 (−0.25, 0.30) | 0.879 |  | 0.04 (−0.19, 0.28) | 0.734 |  | 0.10 (−0.15, 0.35) | 0.424 |
| 21-DOF | 47.23 (−10.42, 104.88) | 0.109 |  | 0.24 (−0.01, 0.49) | 0.065 |  | 0.19 (−0.03, 0.41) | 0.09 |  | 0.14 (−0.09, 0.37) | 0.237 |
| ALD | −14.13 (−83.44, 55.19) | 0.690 |  | 0.04 (−0.26, 0.34) | 0.79 |  | −0.14 (−0.40, 0.12) | 0.292 |  | −0.01 (−0.28, 0.27) | 0.949 |
| DOC | 20.46 (−83.98, 124.90) | 0.701 |  | 0.17 (−0.29, 0.63) | 0.463 |  | 0.03 (−0.36, 0.43) | 0.863 |  | −0.38 (−0.80, 0.03) | 0.068 |
| A4 | −7.50 (−73.62, 58.62) | 0.824 |  | −0.01 (−0.29, 0.28) | 0.973 |  | −0.20 (−0.45, 0.04) | 0.109 |  | −0.06 (−0.32, 0.21) | 0.673 |
| T | −4.99 (−74.52, 64.54) | 0.888 |  | 0.01 (−0.30, 0.31) | 0.97 |  | −0.15 (−0.41, 0.11) | 0.254 |  | −0.11 (−0.38, 0.17) | 0.446 |
| DHT | −17.85 (−82.53, 46.84) | 0.589 |  | −0.01 (−0.30, 0.27) | 0.933 |  | −0.14 (−0.39, 0.10) | 0.244 |  | −0.18 (−0.44, 0.07) | 0.163 |
| DHEA | 15.57 (−47.46, 78.60) | 0.629 |  | 0.04 (−0.24, 0.31) | 0.782 |  | −0.05 (−0.29, 0.19) | 0.685 |  | −0.10 (−0.35, 0.15) | 0.415 |
| DHEAS | −13.06 (−69.52, 43.39) | 0.65 |  | 0.07 (−0.18, 0.32) | 0.577 |  | −0.12 (−0.33, 0.10) | 0.286 |  | −0.11 (−0.34, 0.11) | 0.33 |
| E1 | 20.38 (−30.41, 71.17) | 0.432 |  | 0.15 (−0.07, 0.38) | 0.174 |  | 0.02 (−0.17, 0.22) | 0.799 |  | **−0.21 (−0.41, −0.01)** | **0.037^*^** |
| E2 | 48.35 (−26.12, 122.81) | 0.204 |  | 0.24 (−0.08, 0.57) | 0.147 |  | −0.07 (−0.35, 0.21) | 0.636 |  | −0.15 (−0.45, 0.15) | 0.321 |
| E3 | 12.66 (−11.14, 36.45) | 0.298 |  | 0.06 (−0.05, 0.16) | 0.292 |  | 0.06 (−0.03, 0.15) | 0.196 |  | 0.01 (−0.09, 0.10) | 0.856 |
| P4 | −23.14 (−136.55, 90.26) | 0.689 |  | 0.00 (−0.49, 0.50) | 0.985 |  | −0.27 (−0.70, 0.15) | 0.212 |  | 0.08 (−0.37, 0.54) | 0.713 |
| P5 | −16.18 (−127.15, 94.80) | 0.775 |  | 0.04 (−0.45, 0.52) | 0.876 |  | −0.03 (−0.45, 0.39) | 0.897 |  | 0.00 (−0.44, 0.44) | 0.989 |
| 17-OHP4 | 6.76 (−69.60, 83.13) | 0.862 |  | −0.02 (−0.35, 0.32) | 0.914 |  | −0.06 (−0.35, 0.23) | 0.683 |  | −0.23 (−0.53, 0.07) | 0.14 |
| 17-OHP5 | 22.42 (−8.35, 53.18) | 0.154 |  | 0.06 (−0.08, 0.19) | 0.42 |  | 0.04 (−0.08, 0.15) | 0.525 |  | 0.03 (−0.10, 0.15) | 0.684 |
| Note: Models were adjusted for sampling gestational week, maternal age, education level, pre-pregnancy BMI, delivery mode, parity, HDP, GDM, and infant sex for GA, with GA additionally adjusted for BW, BL, and HC. ^*^*P* < 0.05. | | | | | | | | | | | |

| **Table S10** Sensitivity analyses of associations between steroid hormones (ln-transformed) and ABO, SGA and LGA risk. | | | | | | | | |
| --- | --- | --- | --- | --- | --- | --- | --- | --- |
| Steroid Hormones | ABO | |  | SGA | |  | LGA | |
|  | OR (95% CI) | *P*-value |  | OR (95% CI) | *P*-value |  | OR (95% CI) | *P*-value |
| F | 0.70 (0.37, 1.32) | 0.272 |  | **0.35 (0.13, 0.89)** | **0.027**^*^ |  | 1.28 (0.36, 4.51) | 0.704 |
| E | 0.71 (0.20, 2.57) | 0.603 |  | 0.54 (0.08, 3.79) | 0.531 |  | 1.66 (0.16, 17.32) | 0.672 |
| CORT | 0.71 (0.46, 1.09) | 0.113 |  | 0.58 (0.29, 1.17) | 0.131 |  | 1.15 (0.55, 2.41) | 0.717 |
| 11-DOF | **0.57 (0.35, 0.92)** | **0.022**^*^ |  | **0.45 (0.22, 0.94)** | **0.033**^*^ |  | 0.54 (0.22, 1.32) | 0.177 |
| 21-DOF | 0.69 (0.44, 1.08) | 0.103 |  | 0.57 (0.28, 1.17) | 0.126 |  | 1.14 (0.53, 2.46) | 0.736 |
| ALD | 0.97 (0.58, 1.62) | 0.901 |  | 1.29 (0.57, 2.94) | 0.536 |  | 0.92 (0.35, 2.46) | 0.873 |
| DOC | 0.97 (0.45, 2.07) | 0.931 |  | 0.77 (0.23, 2.56) | 0.667 |  | 1.52 (0.39, 5.97) | 0.548 |
| A4 | **1.95 (1.20, 3.17)** | **0.007**^*^ |  | **2.48 (1.17, 5.23)** | **0.017**^*^ |  | 2.23 (0.96, 5.20) | 0.064 |
| T | **1.95 (1.18, 3.24)** | **0.010**^*^ |  | **2.67 (1.23, 5.80)** | **0.013**^*^ |  | 2.09 (0.90, 4.88) | 0.087 |
| DHT | **1.72 (1.01, 2.91)** | **0.044**^*^ |  | 1.55 (0.71, 3.38) | 0.274 |  | 2.52 (0.94, 6.76) | 0.066 |
| DHEA | 1.24 (0.77, 1.99) | 0.370 |  | 1.35 (0.65, 2.82) | 0.425 |  | 1.37 (0.58, 3.21) | 0.471 |
| DHEAS | 1.18 (0.76, 1.84) | 0.457 |  | 1.70 (0.78, 3.68) | 0.181 |  | 0.76 (0.35, 1.62) | 0.472 |
| E1 | 1.06 (0.72, 1.55) | 0.767 |  | 0.96 (0.55, 1.68) | 0.886 |  | 1.07 (0.54, 2.11) | 0.856 |
| E2 | 1.12 (0.64, 1.96) | 0.698 |  | 1.41 (0.60, 3.35) | 0.430 |  | 1.01 (0.37, 2.78) | 0.979 |
| E3 | **0.81 (0.68, 0.96)** | **0.018**^*^ |  | 0.77 (0.59, 1.01) | 0.056 |  | 0.82 (0.59, 1.14) | 0.232 |
| P4 | 1.12 (0.48, 2.59) | 0.790 |  | 1.76 (0.52, 5.99) | 0.364 |  | 1.01 (0.19, 5.37) | 0.992 |
| P5 | 0.92 (0.41, 2.07) | 0.838 |  | 1.46 (0.42, 5.01) | 0.549 |  | 1.06 (0.25, 4.49) | 0.935 |
| 17-OHP4 | 1.19 (0.67, 2.12) | 0.544 |  | 0.94 (0.44, 1.99) | 0.866 |  | 1.83 (0.61, 5.47) | 0.282 |
| 17-OHP5 | 0.95 (0.75, 1.19) | 0.633 |  | 0.81 (0.57, 1.15) | 0.242 |  | 1.11 (0.74, 1.66) | 0.602 |
| Note: Models were adjusted for sampling gestational week, maternal age, education level, pre-pregnancy BMI, delivery mode, parity, HDP, GDM, and infant sex for ABO, SGA, LGA, and PTB, with GA additionally adjusted for LBW and macrosomia. ^*^*P* < 0.05. | | | | | | | | |

| **Table S11** Sensitivity analyses of associations between steroid hormones (ln-transformed) and LBW, macrosomia, and PTB risk. | | | | | | | | |
| --- | --- | --- | --- | --- | --- | --- | --- | --- |
| Steroid Hormones | LBW | |  | Macrosomia | |  | PTB | |
|  | OR (95% CI) | *P*-value |  | OR (95% CI) | *P*-value |  | OR (95% CI) | *P*-value |
| F | 0.11 (0.01, 2.40) | 0.16 |  | 4.59 (0.56, 37.84) | 0.157 |  | 0.82 (0.28, 2.40) | 0.718 |
| E | 0.08 (0.00, 28.80) | 0.401 |  | 1.83 (0.06, 57.02) | 0.731 |  | 0.85 (0.11, 6.75) | 0.877 |
| CORT | 0.13 (0.01, 1.21) | 0.073 |  | 1.80 (0.65, 4.99) | 0.256 |  | 0.46 (0.19, 1.11) | 0.086 |
| 11-DOF | 0.20 (0.02, 1.76) | 0.148 |  | 0.35 (0.09, 1.40) | 0.137 |  | 0.72 (0.34, 1.52) | 0.391 |
| 21-DOF | 0.06 (0.00, 1.05) | 0.054 |  | 2.10 (0.74, 5.98) | 0.166 |  | 0.46 (0.19, 1.09) | 0.079 |
| ALD | 1.50 (0.11, 20.82) | 0.763 |  | 0.90 (0.20, 3.98) | 0.892 |  | 0.87 (0.37, 2.03) | 0.749 |
| DOC | 0.70 (0.06, 8.68) | 0.784 |  | 0.81 (0.09, 6.96) | 0.844 |  | 1.72 (0.55, 5.40) | 0.351 |
| A4 | 2.28 (0.45, 11.48) | 0.317 |  | 2.20 (0.61, 7.89) | 0.227 |  | 1.79 (0.82, 3.87) | 0.141 |
| T | 3.69 (0.77, 17.58) | 0.102 |  | 2.17 (0.56, 8.48) | 0.264 |  | 1.60 (0.70, 3.68) | 0.269 |
| DHT | 3.82 (0.43, 33.94) | 0.228 |  | 2.54 (0.58, 11.16) | 0.218 |  | 2.04 (0.85, 4.89) | 0.111 |
| DHEA | 3.57 (0.54, 23.73) | 0.187 |  | 1.41 (0.41, 4.83) | 0.589 |  | 1.36 (0.63, 2.91) | 0.433 |
| DHEAS | 2.74 (0.27, 27.61) | 0.391 |  | 1.35 (0.39, 4.65) | 0.634 |  | 1.35 (0.63, 2.89) | 0.436 |
| E1 | 0.61 (0.13, 2.93) | 0.534 |  | 0.71 (0.25, 2.00) | 0.523 |  | 1.32 (0.71, 2.44) | 0.383 |
| E2 | 4.63 (0.27, 79.03) | 0.29 |  | 1.06 (0.24, 4.68) | 0.937 |  | 1.19 (0.49, 2.88) | 0.707 |
| E3 | 0.70 (0.37, 1.32) | 0.275 |  | 0.79 (0.49, 1.29) | 0.353 |  | 0.79 (0.60, 1.03) | 0.083 |
| P4 | 3.98 (0.23, 69.20) | 0.343 |  | 0.11 (0.01, 1.22) | 0.072 |  | 1.31 (0.33, 5.17) | 0.698 |
| P5 | 0.88 (0.06, 11.81) | 0.92 |  | 0.44 (0.06, 3.10) | 0.406 |  | 0.66 (0.18, 2.37) | 0.519 |
| 17-OHP4 | 0.74 (0.09, 5.96) | 0.777 |  | 0.99 (0.17, 5.58) | 0.987 |  | 1.98 (0.76, 5.15) | 0.159 |
| 17-OHP5 | 0.72 (0.29, 1.81) | 0.485 |  | 1.04 (0.58, 1.86) | 0.885 |  | 0.90 (0.62, 1.30) | 0.568 |
| Note: Models were adjusted for sampling gestational week, maternal age, education level, pre-pregnancy BMI, delivery mode, parity, HDP, GDM, and infant sex for ABO, SGA, LGA, and PTB, with GA additionally adjusted for LBW and macrosomia. ^*^*P* < 0.05. | | | | | | | | |

| **Table S12** Sensitivity analyses of associations between steroid hormones (per 1-SD increase) and birth parameters after excluding HDP and GDM . | | | | | | | | | | | |
| --- | --- | --- | --- | --- | --- | --- | --- | --- | --- | --- | --- |
| Steroid Hormones  (SD, ng/mL) | BW (g) | |  | BL (cm) | |  | HC (cm) | |  | GA (week) | |
|  | β (95% CI) | *P*-value |  | β (95% CI) | *P*-value |  | β (95% CI) | *P*-value |  | β (95% CI) | *P*-value |
| F (25.78) | 31.51 (−9.18, 72.20) | 0.130 |  | **0.20 (0.02, 0.38)** | **0.033** |  | 0.14 (−0.02, 0.30) | 0.099 |  | −0.11 (−0.28, 0.06) | 0.200 |
| E (5.62) | 34.84 (−4.67, 74.35) | 0.085 |  | 0.12 (−0.06, 0.30) | 0.206 |  | 0.07 (−0.09, 0.23) | 0.386 |  | −0.06 (−0.23, 0.10) | 0.445 |
| CORT (3.09) | 35.30 (−2.44, 73.04) | 0.068 |  | 0.15 (−0.03, 0.32) | 0.096 |  | 0.13 (−0.02, 0.28) | 0.094 |  | 0.02 (−0.14, 0.18) | 0.781 |
| 11-DOF (0.36) | 21.37 (−19.03, 61.78) | 0.301 |  | −0.03 (−0.21, 0.16) | 0.776 |  | 0.04 (−0.12, 0.20) | 0.592 |  | 0.09 (−0.07, 0.26) | 0.270 |
| 21-DOF (0.16) | 34.83 (−2.83, 72.48) | 0.071 |  | 0.14 (−0.03, 0.31) | 0.111 |  | 0.13 (−0.02, 0.28) | 0.090 |  | 0.03 (−0.13, 0.19) | 0.683 |
| ALD (0.3) | 2.37 (−34.46, 39.21) | 0.900 |  | 0.07 (−0.10, 0.23) | 0.443 |  | −0.08 (−0.22, 0.07) | 0.285 |  | −0.02 (−0.18, 0.13) | 0.774 |
| DOC (0.05) | 10.97 (−31.03, 52.98) | 0.609 |  | 0.04 (−0.15, 0.23) | 0.702 |  | −0.01 (−0.18, 0.15) | 0.897 |  | −0.15 (−0.32, 0.03) | 0.102 |
| A4 (0.78) | 9.87 (−33.10, 52.85) | 0.653 |  | 0.05 (−0.15, 0.24) | 0.632 |  | −0.14 (−0.31, 0.03) | 0.099 |  | −0.08 (−0.26, 0.10) | 0.370 |
| T (0.62) | 14.93 (−29.19, 59.04) | 0.508 |  | 0.07 (−0.13, 0.27) | 0.467 |  | −0.06 (−0.24, 0.11) | 0.476 |  | −0.10 (−0.28, 0.09) | 0.294 |
| DHT (0.09) | 1.75 (−38.56, 42.06) | 0.932 |  | 0.03 (−0.15, 0.21) | 0.742 |  | −0.13 (−0.29, 0.03) | 0.105 |  | **−0.17 (−0.34, −0.01)** | **0.043** |
| DHEA (1.67) | 10.79 (−26.65, 48.24) | 0.573 |  | 0.05 (−0.11, 0.22) | 0.527 |  | −0.04 (−0.19, 0.11) | 0.574 |  | −0.07 (−0.23, 0.08) | 0.364 |
| DHEAS (284.49) | −22.65 (−62.17, 16.87) | 0.262 |  | −0.01 (−0.19, 0.17) | 0.894 |  | −0.15 (−0.30, 0.01) | 0.067 |  | −0.09 (−0.26, 0.07) | 0.261 |
| E1 (1.07) | 17.80 (−21.38, 56.99) | 0.374 |  | 0.06 (−0.11, 0.24) | 0.474 |  | −0.02 (−0.17, 0.14) | 0.831 |  | −0.11 (−0.28, 0.05) | 0.170 |
| E2 (1.95) | 20.57 (−21.38, 62.51) | 0.337 |  | 0.13 (−0.06, 0.32) | 0.194 |  | −0.05 (−0.21, 0.12) | 0.568 |  | −0.09 (−0.27, 0.08) | 0.306 |
| E3 (0.98) | 22.05 (−23.06, 67.15) | 0.339 |  | 0.11 (−0.10, 0.31) | 0.298 |  | 0.09 (−0.09, 0.27) | 0.334 |  | 0.02 (−0.16, 0.21) | 0.798 |
| P4 (10.59) | −13.23 (−53.54, 27.07) | 0.520 |  | −0.04 (−0.22, 0.14) | 0.664 |  | −0.14 (−0.30, 0.02) | 0.089 |  | 0.01 (−0.16, 0.18) | 0.887 |
| P5 (2.75) | −9.97 (−51.35, 31.42) | 0.637 |  | −0.01 (−0.20, 0.18) | 0.928 |  | 0.01 (−0.15, 0.17) | 0.918 |  | −0.02 (−0.19, 0.15) | 0.834 |
| 17-OHP4 (0.95) | 10.84 (−30.29, 51.97) | 0.606 |  | 0.01 (−0.18, 0.19) | 0.934 |  | −0.03 (−0.19, 0.13) | 0.735 |  | −0.15 (−0.32, 0.01) | 0.075 |
| 17-OHP5 (0.64) | 20.22 (−19.39, 59.83) | 0.318 |  | 0.07 (−0.11, 0.25) | 0.472 |  | 0.06 (−0.09, 0.22) | 0.440 |  | 0.12 (−0.05, 0.28) | 0.166 |
| Note: Hormone concentrations were standardized using z-score transformation. Models were adjusted for sampling gestational week, maternal age, education level, pre-pregnancy BMI, delivery mode, parity, HDP, GDM, and infant sex for GA, with GA additionally adjusted for BW, BL, and HC. ^*^*P* < 0.05. | | | | | | | | | | | |

| **Table 13** Sensitivity analyses of associations between steroid hormones (per 1-SD increase) and ABO, SGA, and LGA risk after excluding HDP and GDM. | | | | | | | | |
| --- | --- | --- | --- | --- | --- | --- | --- | --- |
| Steroid Hormones  (SD, ng/mL) | ABO | |  | SGA | |  | LGA | |
|  | OR (95% CI) | *P*-value |  | OR (95% CI) | *P*-value |  | OR (95% CI) | *P*-value |
| F (25.78) | 0.88 (0.66, 1.16) | 0.356 |  | **0.84 (0.72, 0.97)** | **0.021^*^** |  | 1.05 (0.62, 1.78) | 0.850 |
| E (5.62) | 0.92 (0.71, 1.20) | 0.558 |  | 0.88 (0.59, 1.31) | 0.533 |  | 1.07 (0.67, 1.72) | 0.776 |
| CORT (3.09) | 0.84 (0.62, 1.13) | 0.246 |  | 0.69 (0.42, 1.15) | 0.158 |  | 1.09 (0.70, 1.70) | 0.704 |
| 11-DOF (0.36) | **0.70 (****0.50, 0.98)** | **0.037^*^** |  | **0.58 (0.35, 0.95)** | **0.032^*^** |  | 0.53 (0.25, 1.11) | 0.091 |
| 21-DOF (0.16) | 0.85 (0.63, 1.15) | 0.283 |  | 0.73 (0.46, 1.18) | 0.197 |  | 1.10 (0.70, 1.72) | 0.677 |
| ALD (0.3) | 1.11 (0.86, 1.44) | 0.414 |  | 1.22 (0.85, 1.74) | 0.276 |  | 1.03 (0.60, 1.78) | 0.910 |
| DOC (0.05) | 0.96 (0.74, 1.24) | 0.735 |  | 0.83 (0.52, 1.33) | 0.441 |  | 1.10 (0.71, 1.70) | 0.671 |
| A4 (0.78) | **1.36 (****1.07, 1.72)** | **0.012^*^** |  | **1.40 (1.01, 1.94)** | **0.042^*^** |  | 1.42 (0.97, 2.10) | 0.074 |
| T (0.62) | **1.37 (1.08, 1.73)** | **0.009^**^** |  | **1.47 (1.08, 1.99)** | **0.013^*^** |  | 1.32 (0.95, 1.82) | 0.099 |
| DHT (0.09) | **1.32 (1.03, 1.69)** | **0.027^*^** |  | 1.28 (0.90, 1.83) | 0.168 |  | 1.57 (0.99, 2.49) | 0.053 |
| DHEA (1.67) | 1.19 (0.94, 1.53) | 0.154 |  | 1.25 (0.88, 1.78) | 0.208 |  | 1.22 (0.81, 1.85) | 0.341 |
| DHEAS (284.49) | 1.10 (0.85, 1.43) | 0.477 |  | 1.34 (0.90, 1.98) | 0.153 |  | 0.86 (0.51, 1.44) | 0.558 |
| E1 (1.07) | 0.91 (0.67, 1.23) | 0.539 |  | 0.80 (0.50, 1.27) | 0.345 |  | 0.99 (0.58, 1.69) | 0.980 |
| E2 (1.95) | 1.03 (0.77, 1.38) | 0.846 |  | 1.11 (0.74, 1.67) | 0.621 |  | 0.91 (0.51, 1.64) | 0.764 |
| E3 (0.98) | 0.75 (0.53, 1.07) | 0.109 |  | 0.79 (0.49, 1.28) | 0.340 |  | 0.59 (0.28, 1.25) | 0.167 |
| P4 (10.59) | 1.05 (0.80, 1.36) | 0.737 |  | 1.06 (0.72, 1.56) | 0.759 |  | 0.98 (0.57, 1.67) | 0.941 |
| P5 (2.75) | 0.97 (0.75, 1.25) | 0.796 |  | 1.03 (0.71, 1.51) | 0.869 |  | 1.03 (0.66, 1.60) | 0.911 |
| 17-OHP4 (0.95) | 1.00 (0.77, 1.28) | 0.975 |  | 0.81 (0.49, 1.31) | 0.387 |  | 1.17 (0.78, 1.74) | 0.447 |
| 17-OHP5 (0.64) | 0.81 (0.58, 1.13) | 0.211 |  | 0.73 (0.43, 1.23) | 0.232 |  | 1.02 (0.64, 1.63) | 0.929 |
| Note: Hormone concentrations were standardized using z-score transformation. Models were adjusted for sampling gestational week, maternal age, education level, pre-pregnancy BMI, delivery mode, parity, HDP, GDM, and infant sex for ABO, SGA, LGA, and PTB, with GA additionally adjusted for LBW and macrosomia. ^*^*P* < 0.05. | | | | | | | | |
| **Table S14** Sensitivity analyses of associations between steroid hormones (per 1-SD increase) and LBW, Macrosomia, and PTB risk after excluding HDP and GDM. | | | | | | | | |
| Steroid Hormones  (SD, ng/mL) | LBW | |  | Macrosomia | |  | PTB | |
|  | OR (95% CI) | *P*-value |  | OR (95% CI) | *P*-value |  | OR (95% CI) | *P*-value |
| F (25.78) | 0.35 (0.09, 1.39) | 0.136 |  | 1.91 (0.88, 4.14) | 0.101 |  | 0.90 (0.56, 1.42) | 0.639 |
| E (5.62) | 0.54 (0.16, 1.82) | 0.319 |  | 1.11 (0.56, 2.21) | 0.771 |  | 0.93 (0.61, 1.43) | 0.740 |
| CORT (3.09) | 0.08 (0.00, 1.51) | 0.092 |  | 1.40 (0.84, 2.34) | 0.191 |  | 0.66 (0.36, 1.22) | 0.187 |
| 11-DOF (0.36) | 0.25 (0.04, 1.47) | 0.125 |  | 0.48 (0.16, 1.46) | 0.199 |  | 0.81 (0.49, 1.32) | 0.395 |
| 21-DOF (0.16) | 0.08 (0.01, 1.34) | 0.080 |  | 1.47 (0.89, 2.43) | 0.137 |  | 0.66 (0.35, 1.21) | 0.180 |
| ALD (0.3) | 1.13 (0.34, 3.71) | 0.847 |  | 1.23 (0.60, 2.51) | 0.574 |  | 1.11 (0.71, 1.74) | 0.656 |
| DOC (0.05) | 0.83 (0.40, 1.72) | 0.615 |  | 0.83 (0.34, 2.03) | 0.685 |  | 1.05 (0.75, 1.48) | 0.765 |
| A4 (0.78) | 1.07 (0.52, 2.21) | 0.857 |  | 1.34 (0.71, 2.50) | 0.365 |  | 1.27 (0.89, 1.83) | 0.189 |
| T (0.62) | 1.32 (0.75, 2.30) | 0.335 |  | 1.48 (0.77, 2.85) | 0.236 |  | 1.19 (0.81, 1.72) | 0.375 |
| DHT (0.09) | 1.30 (0.57, 2.99) | 0.529 |  | 1.40 (0.67, 2.90) | 0.372 |  | 1.34 (0.91, 1.99) | 0.143 |
| DHEA (1.67) | 1.58 (0.68, 3.63) | 0.285 |  | 1.29 (0.70, 2.35) | 0.412 |  | 1.14 (0.78, 1.68) | 0.490 |
| DHEAS (284.49) | 2.10 (0.60, 7.31) | 0.245 |  | 1.09 (0.54, 2.21) | 0.808 |  | 1.20 (0.79, 1.81) | 0.396 |
| E1 (1.07) | 0.52 (0.13, 2.17) | 0.371 |  | 0.59 (0.17, 2.02) | 0.399 |  | 1.05 (0.66, 1.69) | 0.830 |
| E2 (1.95) | 1.64 (0.54, 4.94) | 0.381 |  | 0.80 (0.33, 1.96) | 0.631 |  | 1.07 (0.66, 1.74) | 0.771 |
| E3 (0.98) | 0.93 (0.30, 2.87) | 0.901 |  | 0.31 (0.07, 1.43) | 0.132 |  | 0.83 (0.47, 1.46) | 0.516 |
| P4 (10.59) | 1.21 (0.60, 2.43) | 0.590 |  | 0.41 (0.15, 1.10) | 0.077 |  | 1.09 (0.73, 1.63) | 0.669 |
| P5 (2.75) | 0.89 (0.47, 1.71) | 0.736 |  | 0.75 (0.38, 1.45) | 0.390 |  | 0.85 (0.56, 1.29) | 0.446 |
| 17-OHP4 (0.95) | 0.75 (0.30, 1.88) | 0.545 |  | 0.86 (0.36, 2.06) | 0.740 |  | 1.11 (0.80, 1.55) | 0.541 |
| 17-OHP5 (0.64) | 0.29 (0.03, 2.45) | 0.253 |  | 1.15 (0.64, 2.07) | 0.631 |  | 0.62 (0.30, 1.29) | 0.202 |
| Note: Hormone concentrations were standardized using z-score transformation. Models were adjusted for sampling gestational week, maternal age, education level, pre-pregnancy BMI, delivery mode, parity, HDP, GDM, and infant sex for ABO, SGA, LGA, and PTB, with GA additionally adjusted for LBW and macrosomia. ^*^*P* < 0.05. | | | | | | | | |

| **Table S15** Sensitivity analyses of QgComp models for associations between maternal steroid hormone mixtures and birth outcomes. | | | | | | | |
| --- | --- | --- | --- | --- | --- | --- | --- |
| Class | Excluding | Sensitivity model OR (95% CI) | | | | | |
|  |  | ABO | SGA | LGA | LBW | Macrosomia | PTB |
| Total mixture | *r*>0.9^a^ | 0.75 (0.40, 1.39) | 0.81 (0.30, 2.18) | 0.48 (0.13, 1.76) | NA | 0.22 (0.01, 4.39) | 0.90 (0.33, 2.48) |
| Androgen | A4 | 1.38 (0.99, 1.93) | **1.75 (1.04, 2.96)** | 1.10 (0.59, 2.03) | 15.34 (0.86, 274.22) | 1.32 (0.54, 3.19) | 1.45 (0.85, 2.48) |
| Total mixture | HDP+GDM | 0.82 (0.42, 1.62) | 0.76 (0.26, 2.26) | 1.00 (0.23, 4.32) | NA | NA | 1.18 (0.37, 3.76) |
| Glucocorticoid | HDP+GDM | 0.81 (0.56, 1.15) | 0.62 (0.36, 1.05) | 0.92 (0.47, 1.80) | 0.01 (0.00, 6.35) | 1.23 (0.38, 4.02) | 0.90 (0.50, 1.65) |
| Mineralocorticoid | HDP+GDM | 1.11 (0.80, 1.55) | 1.19 (0.73, 1.94) | 1.13 (0.64, 2.00) | 4.25 (0.61, 29.66) | 0.90 (0.39, 2.08) | 1.28 (0.75, 2.17) |
| Androgen | HDP+GDM | **1.65 (1.14, 2.41)** | **1.95 (1.10, 3.45)** | 1.26 (0.66, 2.43) | NA | 1.45 (0.59, 3.52) | 1.77 (0.98, 3.21) |
| Estrogen | HDP+GDM | 0.74 (0.50, 1.10) | 0.83 (0.46, 1.49) | 0.80 (0.40, 1.58) | 1.14 (0.02, 62.50) | 0.70 (0.25, 1.97) | 0.84 (0.43, 1.63) |
| Progestogen | HDP+GDM | 1.10 (0.74, 1.65) | 1.01 (0.55, 1.85) | 1.37 (0.69, 2.70) | 1.96 (0.27, 14.06) | 0.53 (0.18, 1.56) | 1.19 (0.63, 2.25) |
| ^a^ CORT was excluded due to its high correlation with 21-DOF (*r* = 0.98). | | | | | | | |
